# A genome-wide association study identifies common variants influencing serum uric acid concentrations in a Chinese population

**DOI:** 10.1186/1755-8794-7-10

**Published:** 2014-02-11

**Authors:** Binyao Yang, Zengnan Mo, Chen Wu, Handong Yang, Xiaobo Yang, Yunfeng He, Lixuan Gui, Li Zhou, Huan Guo, Xiaomin Zhang, Jing Yuan, Xiayun Dai, Jun Li, Gaokun Qiu, Suli Huang, Qifei Deng, Yingying Feng, Lei Guan, Die Hu, Xiao Zhang, Tian Wang, Jiang Zhu, Xinwen Min, Mingjian Lang, Dongfeng Li, Frank B Hu, Dongxin Lin, Tangchun Wu, Meian He

**Affiliations:** 1 Department of Occupational and Environmental Health and the Ministry of Education Key Lab of Environment and Health, School of Public Health, Tongji Medical College, Huazhong University of Science & Technology, Wuhan 430030, Hubei, China; 2 Institute of Urology and Nephrology, First Affiliated Hospital & Center for Genomic and Personalized Medicine, Guangxi Medical University, Nanning, Guangxi 530021, China; 3 Department of Occupational Health and Environmental Health, School of Public Health, Guangxi Medical University, Nanning, Guangxi, China; 4 State Key Laboratory of Molecular Oncology, Cancer Institute & Hospital, Chinese Academy of Medical Sciences and Peking Union Medical College, Beijing 100021, China; 5 Dongfeng Central Hospital, Dongfeng Motor Corporation and Hubei University of Medicine, Shiyan 442008, Hubei, China; 6 Departments of Nutrition and Epidemiology, Harvard School of Public Health, Boston 02115, MA, USA; 7 Department of Epidemiology, School of Public Health and Management, Chongqing Medical University, Chongqing 400016, China

**Keywords:** Genome-wide association study, Serum uric acid, Ethnic differences, Gene-environment interaction

## Abstract

**Background:**

Uric acid (UA) is a complex phenotype influenced by both genetic and environmental factors as well as their interactions. Current genome-wide association studies (GWASs) have identified a variety of genetic determinants of UA in Europeans; however, such studies in Asians, especially in Chinese populations remain limited.

**Methods:**

A two-stage GWAS was performed to identify single nucleotide polymorphisms (SNPs) that were associated with serum uric acid (UA) in a Chinese population of 12,281 participants (GWAS discovery stage included 1452 participants from the Dongfeng-Tongji cohort (DFTJ-cohort) and 1999 participants from the Fangchenggang Area Male Health and Examination Survey (FAMHES). The validation stage included another independent 8830 individuals from the DFTJ-cohort). Affymetrix Genome-Wide Human SNP Array 6.0 chips and Illumina Omni-Express platform were used for genotyping for DFTJ-cohort and FAMHES, respectively. Gene-environment interactions on serum UA levels were further explored in 10,282 participants from the DFTJ-cohort.

**Results:**

Briefly, we identified two previously reported UA loci of *SLC2A9* (rs11722228, combined *P* = 8.98 × 10^-31^) and *ABCG2* (rs2231142, combined *P* = 3.34 × 10^-42^). The two independent SNPs rs11722228 and rs2231142 explained 1.03% and 1.09% of the total variation of UA levels, respectively. Heterogeneity was observed across different populations. More importantly, both independent SNPs rs11722228 and rs2231142 were nominally significantly interacted with gender on serum UA levels (*P* for interaction = 4.0 × 10^-2^ and 2.0 × 10^-2^, respectively). The minor allele (T) for rs11722228 in *SLC2A9* has greater influence in elevating serum UA levels in females compared to males and the minor allele (T) of rs2231142 in *ABCG2* had stronger effects on serum UA levels in males than that in females.

**Conclusions:**

Two genetic loci (*SLC2A9* and *ABCG2*) were confirmed to be associated with serum UA concentration. These findings strongly support the evidence that *SLC2A9* and *ABCG2* function in UA metabolism across human populations. Furthermore, we observed these associations are modified by gender.

## Background

Uric acid (UA) is the primary end-product of purine metabolism in human beings. Most of the UA is derived from the metabolism of endogenous purine including cell turnover and synthesis. The UA excretion and reabsorption is mostly in kidney [1,2]. The UA concentration in human blood is more than fifty times higher than that in other mammals, because in most of the animals UA could be further catalyzed to allantoin by urate oxidase or uricase (the copper-binding enzyme) [1,3]; however, human beings lack uricase and have higher UA levels which could result in hyperuricemia and gouty arthritis [4]. UA could serve as an antioxidant by removing singlet oxygen and radicals [5]. However, elevated UA concentration can lead to a variety of disorders, including gout, hypertension, metabolic syndrome, diabetes mellitus, and cardiovascular disease [6-10].

It is indicated that the conventional factors including age, body mass index (BMI), alcohol consumption, and cigarette smoking could influence serum UA concentrations [11-16]. In addition, serum UA levels were also determined by genetic factors with heritability ranged from 25% to 63% [17-19]. Recent GWASs have identified multiple loci of *ABCG2*, *SLC2A9*, *SLC17A1*, *SLC16A9*, *SLC22A11*, *GCKR*, *PDZK1*, *GCKR*, *RREB1*, *LRRC16A*, *WDR1*, *TRIM46*, *INHBB*, *SFMBT1*, *TMEM171*, *VEGFA*, *BAZ1B*, *PRKAG2*, *STC1*, *HNF4G*, *A1CF*, *ATXN2*, *UBE2Q2*, *IGF1R*, *NFAT5*, *MAF*, *HLF*, *ACVR1B-ACVRL1*, and *B3GNT4* associated with UA levels in European [20-26]. However, only two GWA studies have been conducted in Asians [26,27]. In addition, previous study indicated that the serum UA concentration was influenced by gene-environmental interactions [22]. In order to investigate the genetic determines of serum UA in Asians, especially in Chinese, we conducted a GWA study in 3,451 individuals, and subsequently replicated the top SNPs in additional 8,830 healthy participants. In addition, we further examined whether the top SNPs interacted with gender, BMI, alcohol drinking, and cigarette smoking in determining serum UA levels respectively.

## Methods

### Study participants

In the discovery stage, we performed a GWAS of two studies in the Chinese Han population: the DFTJ-cohort consisted of 1,461 healthy individuals and the FAMHES included 2,012 Han healthy individuals aged 20 to 69 years old. The DFTJ cohort [28] and the FAMHES [29] were described in detail elsewhere. All of the participants included in the GWAS stage were recruited at health check-ups without chronic diseases such as cardiovascular disease and cancer. Briefly, the DFTJ-cohort initiated in 2008 is a long-term prospective, population-based cohort study designed to determine the gene-environmental interaction on several chronic diseases (obesity, diabetes mellitus, cardiovascular disease, etc.) and cancer in employees from Dongfeng Motor Corporation (DMC). The FAMHES was launched in 2009 in Fangchenggang city, Guangxi, southwest China in 2009 and enrolled 4,303 Chinese men with 17 to 88 years-old; this study was designed to examine the genetic, environmental, and their interactions on the development of age-related chronic diseases. The 8,830 healthy individuals included in the validation stage were selected from the DFTJ-cohort excluding the initial 1,461 subjects and had no diagnosed chronic diseases such as cardiovascular disease, cancer, and gout et al.

The detailed information about the GWAS population and the replication samples is shown in (Additional file 1: Table S1). All the participants provided written informed consent and the ethical committees in the Tongji Medical College and Guangxi Medical University approved this research project.

### Measurement of serum UA levels and the covariates

A baseline physical examination was conducted and demographic information was collected via standard questionnaire. Overnight fasting venous blood specimens were obtained and serum UA levels were measured by the ARCHITECT Ci8200 automatic analyzer (ABBOTT Laboratories. Abbott Park, Illinois, U.S.A) using the Abbott Diagnostics reagents following the manufacturer’s instructions in the DFTJ cohort [28] and using automatic analyzer (Dade Behring, USA) with original reagents in the FAMHES study. Weight and standing height were measured with light indoor clothing and in bare feet. Those who had smoked at least one cigarette per day for more than half a year either currently or formerly were defined as smokers; otherwise they were viewed as non-smokers. Alcohol drinking was divided into two categories: drinkers and non-drinkers. Those who had drunk at least once a week for more than half a year either currently or formerly were defined as drinkers; otherwise they were viewed as non-drinkers.

### Sample genotyping and quality control

We performed the GWAS scan in 1,461 subjects from the DFTJ-cohort using Affymetrix Genome-Wide Human SNP Array 6.0 chips following the manufacture’s protocol. Totally, we genotyped 906,703 SNPs among 1,461 subjects. After stringent QC filtering individuals with genotyping call rate < 95% were excluded (9 subjects) for further analysis. SNPs were excluded when 1) MAF < 0.01; 2) Hardy-Weinberg Equilibrium (HWE) test *P*-value < 0.0001; 3) SNPs call rate < 95%. Finally, 658,288 SNPs in 1,452 subjects with an overall call rate of 99.68% were used for further analysis.

We used the Illumina Omni-Express platform to carry out the GWAS scan in FAMHES. There were 1,999 individuals (sample call rate >95%) included in the final statistical analysis. Based on quality control criteria, SNPs were excluded when *P* < 0.001 for the HWE test, MAF < 0.01, or genotype call rate < 95%. Finally, 709,211 SNPs were kept for further analysis.

In the validation stage, ten SNPs were selected based on the following criteria: 1) SNP with *P* < 1.0 × 10^-5^ for all GWAS samples; 2) when multiple SNPs showed a strong LD (r^2^ ≥ 0.8), SNPs previously reported in the literature were prior selected; 3) Clear genotyping clusters; 4) MAF ≥ 0.05. We used the iPLEX system (Sequenom) and/or the TaqMan assay (Applied Biosystems) [30,31] to genotype the 10 SNPs. The primers and probes were available upon request.

### Imputation

We performed ungenotyped SNPs imputation using MACH 1.0 software (see URLs) via LD information from the HapMap phase II database (CHB + JPT as reference set, 2007-08_rel22, released 2007-03-02) in the DFTJ-cohort GWAS. Genotyped SNPs in the FAMHES GWAS were inferred using the IMPUTE program [32], and the reference panels used for imputation in the FAMHES study were HapMap rel.24, build 36, CHB + JPT. The Imputed SNPs with high genotype information content (proper info > 0.5 for IMPUTE and Rsq > 0.3 for MACH) were retained for the further association analysis. Finally, 2,468,160 SNPs were used for the further analysis.

### Statistical analysis

Analysis for serum UA was performed on natural logarithmic (ln)-transformed values because of skewed distributions. Genome-wide association tests were performed using the additive model by linear regression analysis with adjustment for age, gender, BMI, cigarette smoking, and alcohol drinking implement using PLINK1.06 [33]. The top two eigenvectors were also adjusted as covariates in the linear regression analysis. Population structure was evaluated using principal components analysis (PCA), as implemented by EIGENSTRAT software [34] and quantile-quantile (QQ) plot was generated by using R 2.11.1 (see URLs). Heterogeneity among the study populations was evaluated by the I^2^ statistic [35]. The Manhattan plot of -log10 P, LD structures and haplotype block plots were generated by using Haploview (v4.1) [36]. The association studies with the imputation data were performed using the ProbABEL software [37]. The meta-analysis of the DFTJ-cohort GWAS data and the FAMHES GWAS data was performed using a fixed-effects meta-analysis with inverse variance weighted method using the metal software [38]. The regional association plots were drawing using SNAP software [39].

The power of the present study was calculated using the Quanto software package [40]. We used the mean of serum UA 292.5 μmol/L, MAF values obtained from the combined genotype dataset, and assumed an additive genetic model with α = 0.05 in two tail tests to calculate the statistical power.

A conditional analysis was done in linear regression model to examine the independence of the four associated SNPs in the combined data (partial r^2^ indicated the proportion of the serum UA variation explained by each SNP). We did the conditional analysis via including the four significant SNPs in the linear regression model adjusting for age, gender, BMI, cigarette smoking, and alcohol drinking in 10,282 individuals from the DFTJ-cohort. The SNPs with *P* value < 0.05 remained in the multivariate model were considered to be associated with the serum UA levels independently.

The combined dataset including the DFTJ-cohort GWAS data and the validation stage data totaling 10,282 individuals used in gene-environment interaction analysis was tested by introducing the interaction terms (SNP × gender, SNP × BMI, SNP × alcohol drinking, and SNP × cigarette smoking) into the model, adjusting for the covariates including age, gender, BMI, alcohol drinking, and cigarette smoking. The *P* value less than 0.05 for the interaction term was considered statistical significant. Considering multiple interaction tests were conducted, we further did the multiple test [41] for the gene-environmental interaction analysis. All statistical analyses were performed with SPSS (version 15.0; SPSS, Chicago, IL), and SAS version 9.2 (SAS Institute, Cary, NC, USA).

## Results

### Genome-wide association of serum UA levels

**Figure 1:**
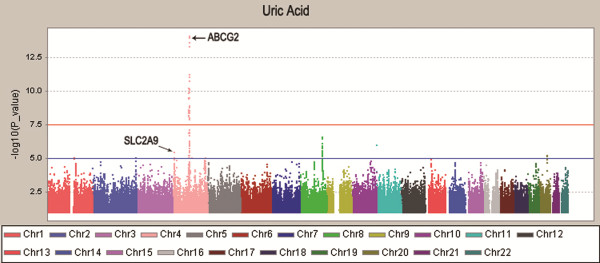
**Manhattan plot of genome–wide association analyses for serum uric acid concentrations.** The X-axis shows chromosomal positions. Y-axis shows –log_10_*P*-values. The red line indicates the genome-wide significance level of 5.0 × 10^-8^ and the blue horizontal line corresponds to a *P* value of 1.0 × 10^-5^.

The demographics of the participants are displayed in (Additional file 1: Table S1). The Q-Q plot revealed no inflation of type I error rate due to population stratification, with a genomic control inflation factor of 1.007 (Additional file 2: Figure S1). No heterogeneity was observed for the SNPs presented in the Table 1 between the DFTJ-cohort study and the FAMHES. As the Manhattan plot (Figure 1) and the regional association plots (Additional file 3: Figure S2) indicated, at the discovery stage the *ABCG2* was significantly associated with the serum UA at a genome-wide significance level.

**Table 1:** Genome-wide association analyses for serum uric acid levels

**SNP**	**Chr**	**bp**	**Genes**	**Locus**	**Minor/major allele**	**GWAS(n = 3,451)**			**Validation(n = 8,830)**			**Combined(n = 12,281)**
						**MAF**	**Effect size (s.e.m.)**	***P***- **value**^**a**^	**MAF**	**Effect size (s.e.m.)**	***P***^-^**value**^**a**^	***P- *** **value**^**a**^
rs11722228	4	9524839	*SLC2A9*	intron	T/C	0.31	0.028 (0.006)	3.68 × 10^-6^	0.31	0.043 (0.004)	4.16 × 10^-26^	8.98 × 10^-31^
rs4148152	4	89279933	*ABCG2*	intron	C/T	0.35	−0.029 (0.006)	1.22 × 10^-7^	0.33	−0.029 (0.004)	4.49 × 10^-13^	2.95 × 10^-18^
rs3114018	4	89283605	*ABCG2*	intron	T/G	0.38	−0.034 (0.006)	2.75 × 10^-9^	0.38	−0.029 (0.004)	1.02 × 10^-12^	4.49 × 10^-20^
rs2231142	4	89271346	*ABCG2*	exon	T/G	0.29	0.046 (0.006)	1.19 × 10^-14^	0.31	0.045 (0.004)	1.01 × 10^-25^	3.34 × 10^-42^

The NCBI build 36 was used as the reference genome. Chr, chromosome. MAF indicates the minor allele frequency calculated using the data from all the subjects in the analysis. Effect size, represents the effect of a minor allele on the standardized trait (estimated coefficient of the term for the number of the minor alleles).

^a^Linear regression analysis adjusted for age, sex, body mass index (BMI), cigarette smoking, and alcohol drinking.

Totally we further investigated ten SNPs in the validation stage and four common variants were associated with serum UA levels at a genome-wide significant level of 5 × 10^-8^: *SLC2A9* (rs11722228, combined *P* = 8.98 × 10^-31^) and *ABCG2* (Combined *P* = 3.34 × 10^-42^ for rs2231142) (Table 1). Both loci of *ABCG2* and *SLC2A9* have been reported previously [20,22,27,42]. If SNPs are in the same locus, we chosen r^2^ of SNPs less than 0.8 for further investigating (r^2^ = 0.168 for rs2231142 and rs4148152; r^2^ = 0.224 for rs2231142 and rs3114018; r^2^ = 0.669 for rs4148152 and rs3114018). (Additional file 3: Figure S2). However, we failed to replicate the top SNPs in the other loci of *SEC22B*, *CENTG2, TET2, VEGFC*, and *TNFRSF11B* in the validations stage (Additional file 4: Table S2).

The SNP rs11722228 in *SLC2A9* and rs2231142 in *ABCG2* were independently associated with serum UA levels and accounted for 1.03% and 1.09% of the serum UA variance respectively (Additional file 5: Table S3).

### Ethnic differences in major genetic variants associated with serum UA levels

We compared the results in the present study with those in Japanese and Europeans. As Table 2 showes, the effect sizes of most of these loci showed consistent direction across the populations. *ABCG2* was associated with serum UA levels across different populations. We found significant association of serum UA with SNP rs4148152 in *ABCG2* in our study (combined *P* = 2.95 × 10^-18^). However, till now we did not find any reports of this SNP in European populations, which might be attributable to very low MAF (0.017) in Europeans. Furthermore, the SNP rs4148152 was in moderate LD with the SNP rs311408 in the present study (r^2^ = 0.669) but in very low LD in Europeans (r^2^ = 0.046; HapMap CEU). SNP rs12356193 in *SLC16A9*, rs10480300 in *PRKAG2* and rs653178 in *ATXN2* were significantly associated with serum UA in European populations but were monoallelic in Asians. Similar findings were found for loci of *SLC2A9* (rs16890979 and rs734553), *SLC22A11* (rs17300741), and recently reported new loci of *TRIM46* (rs11264341), *VEGFA* (rs729761), *BAZ1B* (rs1178977), *STC1* (rs1778674), *A1CF* (rs10821905), *UBE2Q2* (rs1394125), and *HLF* (rs7224610) [25] which were associated with serum UA in Europeans, however, these associations were not replicated in Asians (Japanese and Chinese in the present study). This might be due to the very lower MAF in Asians. However, the relative small sample size in the present study limited us to detect these associations with enough power. In addition, we failed to replicate the SNPs rs742132 in *LRRC16A*, rs780094 in *GCKR*, rs17632159 in *TMEM171*, rs17050272 in *IINHBB*, and rs7188445 in *MAF* which were reported in European [20,25], same as the findings in the Japanese population, albeit the MAFs of both of the SNPs are similar between Asians and Europeans, suggesting that there were genetic discrepancy on serum UA levels among different ethnic groups. The moderate effect size of these loci on serum UA levels and the limited sample size in the present study might be another potential explanation.

**Table 2:** Ethnic differences in major genetic variants associated with serum uric acid levels

					**Our study **^**a**^**(Chinese, discovery stage)**			**Japanese**			**European ancestry**		
**Gene**	**SNP**	**Chr**	**Position**	**Location**	**MAF (allele)**	**Effect size**	***P-*** **value**	**MAF (allele)**	**Effect size**	***P-*** **value**	**MAF (allele)**	**Effect size**	***P-*** **value**
*PDZK1*	rs12129861	1	144437046	5′-	0.20(A)	−0.011	0.074	0.087(A)	--	--	0.46(A)	−0.062 [20]	2.68 × 10^-9^
*TRIM46*	rs11264341	1	153418117	Intron	0.33(C)	0.017	4.49 × 10^-3^	0.24(C)	0.074 [25]	9.5× 10^–4^	0.43(T)	−0.050 [25]	6.2 × 10^–19^
*INHBB*	rs17050272	2	121022910	N/A	0.45(A)	0.008	0.174	0.49(A)	0.033 [25]	4.4× 10^–2^	0.43(A)	0.035 [25]	1.6 × 10^–10^
*GCKR*	rs780094	2	27594741	Intron	0.44(T)	0.0133	0.014	0.43(T)	0.036 [27]	5.12 × 10^-6^	0.42(T)	0.050 [20]	1.40 × 10^-9^
*LRP2*	rs2544390	2	169913092	Intron	0.49(C)	−0.001	0.73	0.49(C)	−0.082 [27]	3.74 × 10^-8^	0.37(T)	--	--
*SFMBT1*	rs6770152	3	53075254	N/A	0.36(T)	−0.010	0.071	0.46(T)	−0.016 [25]	0.31	0.42(G)	0.044 [25]	2.6 × 10^–16^
*SLC2A9*	rs11722228	4	9524839	Intron	0.31(T)	0.028	3.68 × 10^-6^	0.45(T)	0.164 [27]	7.09 × 10^-24^	0.50(T)	0.167 [20]	1.75 × 10^-75^
*SLC2A9*	rs16890979	4	9531265	Exon	0.02(T)	−0.024	0.285	0.01(T)	−0.178 [27]	3.07 × 10^-2^	0.29(T)	−0.340 [20]	3.55 × 10^-189^
*SLC2A9*	rs734553	4	9532102	Intron	0.02(C)	−0.023	0.286	0.006(C)	--	--	0.23(C)	0.315 [20]	5.22 × 10^-201^
*ABCG2*	rs3114018	4	89283605	Intron	0.38(T)	−0.034	2.75 × 10^-9^	0.26(A)	--	--	0.48(G)	−0.057 [20]	2.93 × 10^-12^
*ABCG2*	rs2231142	4	89271347	Exon	0.29(T)	0.046	1.19 × 10^-14^	0.31(T)	0.121 [27]	1.62 × 10^-13^	0.11(T)	0.173 [20]	3.10 × 10^-26^
*ABCG2*	rs4148152	4	89279933	Intron	0.35(C)	−0.029	1.22 × 10^-7^	0.19( C)	--	--	0.017(C)	--	--
*ABCG2*	rs4148155	4	89273691	Intron	0.29(A)	−0.046	9.23 × 10^-15^	0.30(G)	0.121 [27]	1.14 × 10^-13^	0.11(A)	−0.170 [20]	1.50 × 10^-25^
*TMEM171*	rs17632159	5	72467238	N/A	0.31(C)	−0.009	0.129	0.28(C)	−0.045 [25]	0.011	0.31(C)	−0.039 [25]	3.5 × 10^–11^
*VEGFA*	rs729761	6	43912549	N/A	0.14(T)	−0.013	0.092	0.07(T)	0.002 [25]	0.97	0.30(T)	−0.047 [25]	8.0 × 10^–16^
*LRRC16A*	rs742132	6	25715550	Intron	0.25(A)	−0.001	0.8705	0.31(A)	0.008 [27]	0.609	0.30(A)	0.054 [20]	8.50 × 10^-9^
*SLC17A1*	rs1183201	6	25931423	Intron	0.18(A)	−0.009	0.2	0.16(A)	−0.063 [27]	1.73 × 10^-3^	0.48(A)	−0.062 [20]	3.04 × 10^-14^
*SLC17A3*	rs1165205	6	25978521	Intron	0.19(T)	−0.010	0.139	0.16(T)	−0.07 [27]	5.04 × 10^-4^	0.48(T)	0.060 [20]	1.60 × 10^-13^
*PRKAG2*	rs10480300^***b***^	7	151036938	Intron	0.00(T)	--	--	0.00(T)	--	--	0.28(T)	0.035 [25]	4.1 × 10^–9^
*BAZ1B*	rs1178977	7	72494985	Intron	0.08(G)	0.011	0.291	0.11(G)	−0.058 [25]	0.025	0.19(G)	−0.047 [25]	1.2 × 10^–12^
*STC1*	rs17786744	8	23832951	N/A	0.35(G)	0.0001	0.989	0.25(G)	0.018 [25]	0.32	0.42(G)	0.029 [25]	1.4 × 10^–8^
*HNF4G*	rs2941484	8	76641323	3utr	0.32(T)	0.024	1.7× 10^-5^	0.43(T)	0.050 [25]	1.8× 10^-3^	0.44(T)	0.044 [25]	4.4 × 10^–17^
*A1CF*	rs10821905	10	52316099	5′near gene	0.04(A)	0.008	0.584	0.05(A)	0.075 [25]	0.042	0.18(A)	0.057 [25]	7.4 × 10^–17^
*SLC16A9*	rs12356193^*b*^	10	61083359	Intron	0.00(C)	--	--	0.00(C)	--	--	0.18(C)	0.080 [20]	1.07 × 10^-8^
*SLC22A11*	rs17300741	11	64088038	Intron	0.07(A)	0.018	0.087	0.03(A)	0.063 [27]	0.197	0.49(A)	0.060 [20]	6.68 × 10^-14^
*SLC22A12*	rs505802	11	64113648	Intron	0.23(T)	−0.008	0.196	0.18(T)	−0.231 [27]	1.00 × 10^-31^	0.30(T)	−0.060 [20]	2.04 × 10^-9^
*SLC22A12*	rs506338	11	64197496	Intron	0.23(C)	−0.005	0.502	0.17(C)	−0.229 [27]	2.34 × 10^-31^	0.29(T)	--	--
*ATXN2*	rs653178^***b***^	12	110492139	Intron	0.00(C)	--	--	0.00(C)	--	--	0.49(C)	0.035 [25]	7.2 × 10^–12^
*UBE2Q2*	rs1394125	15	73946038	Intron	0.008(A)	0.014	0.188	0.08(A)	0.021 [25]	0.48	0.34(A)	0.043 [25]	2.5 × 10^–13^
*IGF1R*	rs6598541	15	97088658	Intron	0.41(A)	0.015	9.85× 10^-3^	0.50(A)	0.033 [25]	0.038	0.36(A)	0.043 [25]	4.8 × 10^–15^
*NFAT5*	rs7193778	16	68121391	N/A	0.05(C)	0.004	0.769	0.10(C)	0.053 [25]	0.048	0.14(C)	0.046 [25]	8.2 × 10^–10^
*MAF*	rs7188445	16	78292488	N/A	0.27(A)	−0.003	0.592	0.31(A)	−0.06 [25]	4.5× 10^-4^	0.33(A)	−0.032 [25]	1.6 × 10^–9^
*HLF*	rs7224610	17	50719787	Intron	0.13(C)	0.004	0.579	0.18(C)	0.004 [25]	0.83	0.42(C)	0.042 [25]	5.4 × 10^–17^

The NCBI build 36 was used as the reference genome. ^a^Sample size, 3451, the P values in Han Chinese population were from meta-analysis of two GWASs (DFTJ-cohort and FAMHES) in discovery stage; --, information is not available. ^**b**^These SNP is monoallelic in HapMap CHB and HapMap JPT.

### Interaction of SNPs with gender, BMI, cigarette smoking, and alcohol drinking

**Figure 2:**
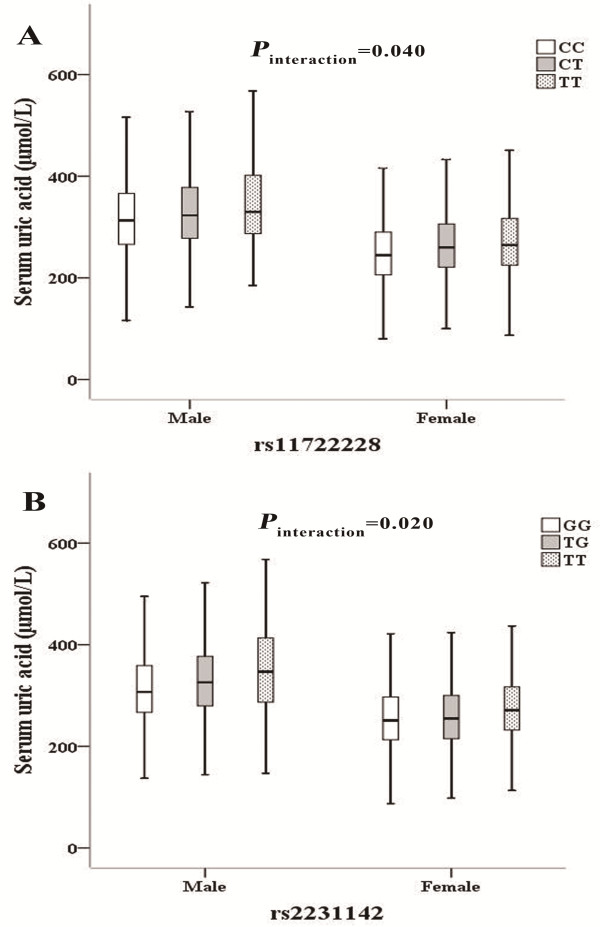
**Gene-gender interactions for *SLC2A9 *rs11722228 and *ABCG2 *rs2231142.** The *P*-values were calculated by using natural log transformed uric acid concentrations as dependent variable. The Y axis represented mean of uric acid concentrations. The X axis represented different gender groups. Multivariate adjusted model was used to analyze the **(A)** interaction for *SLC2A9* rs11722228 and gender in determining uric acid concentration, **(B)** interaction for *ABCG2* rs2231142and gender in determining uric acid concentration.

We further explored the interactions between the two independently-associated SNPs (rs11722228 in *SLC2A9* and rs2231142 in *ABCG2*) and gender, BMI, cigarette smoking, and alcohol drinking on serum UA levels in 10,282 individuals from the DFTJ-cohort. Both SNPs rs11722228 and rs2231142 were nominally interacted with gender on serum UA levels (*P* = 0.04 and *P* = 0.02, respectively; Additional file 6: Table S4 and Figure 2). The minor allele (T) for rs11722228 in *SLC2A9* has greater influence in elevating serum UA levels in females compared to males (beta = 0.051 in females vs. beta = 0.035 in males), similar with the previously reported results [20]. In contrast, for SNP rs2231142 in *ABCG2*, the minor allele (T) had stronger effects on serum UA levels in males than those in females (beta = 0.037 in females vs. beta = 0.057 in males), consistent with the findings in the European populations and African Americans [20,22]. However, the gene-gender interaction altered to null after the multiple test based on the FDR approach. Besides, the rs11722228 accounted for 1.33% and 0.98% and rs2231142 accounted for 0.93% and 2.25% of the total variance of the serum UA levels for females and males, respectively.

In addition, the SNP rs11722228 was also interacted with alcohol drinking and cigarette smoking (*P* for interaction = 0.016 and 0.035, respectively; Additional file 6: Table S4). Considering that the SNP rs11722228 had different effect size on serum UA levels in males and females and in the present study 95.1% smokers and 86.7% drinkers were males, we restricted our analysis in males. However, we failed to detect the interactions between rs11722228 and cigarette smoking and alcohol drinking on serum UA levels anymore (data not showed), suggesting that the SNP-smoking and the SNP-drinking interaction was driven by the gender differences in the serum UA concentrations.

## Discussion

In this two-stage GWAS, we replicated two previously reported loci (*ABCG2* and *SLC2A9*) associated with serum UA levels and found significant gene-gender interactions in Chinese Han population. In addition, ethnic differences were observed between Asian and European populations.

*SLC2A9* is located in chromosome 4p16-15.3 and encodes glucose transporter 9 (GLUT9) which can reabsorb UA in renal tubules [4]. Several studies have reported the association between *SLC2A9* and serum UA levels [20,27,43,44]. Importantly, *SLC2A9* is a transporter for both fructose and urate [45]. Fructose intake could facilitate UA formation in liver via increasing purine breakdown. In addition, animal evidences indicated a causal relationship between fructose intake, serum UA, and metabolic syndrome [11,46,47].

*ABCG2* is an UA exporter that mediates urate excretion in the kidney. Multiple evidences indicated that the common variants in *ABCG2* could reduce the transport function and result in the hyperuricemia and gout [44,48,49]. In the present study, the missense SNP rs2231142 in *ABCG2* showed the strongest association with serum UA level. This missense SNP could result in a glutamine-to-lysine amino acid substitution and the glutamine residue is highly conserved across species and the LD pattern differs in Chinese population and European population.

Comparisons of the SNPs of the association studies for serum UA in different populations are of great interest. The present study replicated two previously reported loci of *SLC2A9* and *ABCG2* associated with serum UA levels. SNP rs11722228 in *SLC2A9* explained 1.03% variation compared to 1.33% in Japanese population [27] and rs2231142 in *ABCG2* accounted for 1.09% of the variation of serum UA levels in our study compared to 1.20% in white individuals and 0.30% in African Americans [22]. Previous studies reported that variants in *SLC2A9* show the strongest effect on serum uric acid levels compared to *ABCG2*[20,22]. However, in the present study we observed that the loci of *ABCG2* and *SLC2A9* show comparable amounts of explained variance (1.09% and 1.03% respectively). Table 2 showed that there was a little difference in MAFs in both variants rs2231142 located in *ABCG2* and rs11722228 located in *SLC2A9* (0.29 and 0.31) in Chinese which were different from that observed in Europeans (0.11 and 0.50 respectively).

Owing to the SNPs of rs12356193 in *SLC16A9*, rs10480300 in *PRKAG2*, and rs653178 in *ATXN2* are monoallelic in Asians, we failed to replicate the SNPs of rs12356193, rs10480300 and rs653178 those were identified in Europeans. In addition, we found notable differences in MAFs for rs16890979 in *SLC2A9*, rs17300741 in *SLC22A11*, rs10821906 in *AICF*, rs1394125 in *UBE2Q2*, rs7193778 in *NFAT5* and rs7224610 in *HLF* (0.02 , 0.07, 0.04, 0.008, 0.05, 0.13 respectively for Chinese population and 0.29, 0.49, 0.18, 0.34, 0.14, 0.42 respectively for European populations) Besides, both rs16890979 (r^2^ = 0.005) and rs734553 (r^2^ = 0.005) in the gene *SLC2A9* were in very low LD with the SNP rs11722228 in Asians compared to that in European populations (r^2^ = 0.202 for rs16890979; r^2^ = 0.193 for rs734553). The very low MAF and the difference in the LD structure might partly explain the discrepancy of the associations between the Europeans and Asians. In addition, relative small sample size in the present study and the moderate effect size of the loci on serum UA levels also limited us to have enough power to detect these associations.

Serum UA levels are lower in females than that in males. More importantly, gene-gender interactions were observed for the two independent SNPs of rs11722228 and rs2231142. The minor allele (T) for rs11722228 has greater influences in elevating serum UA levels in females compared to males, consistent with the previous study [23]. For rs2231142, the TT genotype was related to higher UA as compared with GG and GT genotype in both males and females. This might be due to that the rs2231142 T allele was related to the reduced ability to excrete UA [50]. In addition, the minor allele T allele of rs2231142 has greater effects on the serum UA levels in males than those in females (*P* for interaction = 0.02), in consistent with the findings from the Europeans [22]. This gender difference might be due to the specific physiological characteristics in females. It is also reported that the estrogens might increase the renal clearance of serum UA [51], however, the mechanism underlying the gene-gender interaction remains to be further elucidated.

To our best of knowledge, this is the first GWA study on serum UA levels in Chinese population. Because of the relative small sample size in the discovery stage, we might have limited power to detect the associations of the SNPs with small effect size and/or low MAF. However, in the present study we identified two reported loci (*SLC2A9* and *ABCG2*) associated with serum UA levels, suggesting that our study was capable of identifying significant loci associated with serum UA levels. In addition, the combined data in our study has more than 90% statistic power to detect the interaction between SNP rs11722228 and gender; 70.4% statistical power to detect the interaction between SNP rs2231142 and gender on serum UA levels. Furthermore, our study confirmed the gene-gender interaction on serum UA levels and observed that the *ABCG2* and *SLC2A9* functioned differently in males and females across different populations [20].

## Conclusions

Our study replicated two loci of *SLC2A9* and *ABCG2* associated with serum UA levels in a Chinese Han population. Heterogeneity was observed among different ethnic populations. In addition, we found both the two loci interacted with gender on serum UA levels. These two loci had different effects on serum UA levels in males and females. Further studies are needed to validate our findings and investigate the underlying mechanisms of the gene-gender interaction.

## Competing interests

The authors declare that they have no competing interests.

## Authors’ contributions

Projects conception: WTC, MZN, HMA, FBH. Study design: WTC, HMA, YBY, LDX, WC, YHD, ZXM, YJ, GH. Sample processing and database establishment: YBY, YXB, HYF, ZL, DQF, HSL, ZJ, MXW, LMJ, LDF, QGK, GL, HD, ZX, FYY, and WT. Genotyping: HMA, YBY, And GLX. Imputation: HMA, YBY, GLX. Data analysis: YBY, DXY, and LJ. Interpretation of the data: HMA, MZN, YBY. Drafted the manuscript: YBY and MZN. Revised manuscript: WTC, HMA, MZN. All authors have read and approved of the final version of this manuscript.

## Pre-publication history

The pre-publication history for this paper can be accessed here:

http://www.biomedcentral.com/1755-8794/7/10/prepub

## Supplementary Material

Additional file 1: Table S1 Characteristics of the subjects participated in this study. Click here for file

Additional file 2: Figure S1 Q-Q plots for QTL analyses. Click here for file

Additional file 3: Figure S2 Associations of SNPs in Table 2 with serum uric acid concentrations. Click here for file

Additional file 4: Table S2 SNPs failed to be validated in the validation stage in the present study. Click here for file

Additional file 5: Table S3 Covariates and SNPs in relation to serum uric acid levels in 10,282 individuals. Click here for file

Additional file 6: Table S4 Interaction between SNPs and gender, BMI, alcohol drinking and cigarette smoking. Click here for file

## URLs

R statistical environment, http://cran.r-project.org/;

PLINK, http://pngu.mgh.harvard.edu/~purcell/plink/;

MACH 1.0, http://www.sph.umich.edu/csg/abecasis/mach/;

International HapMap Project, http://www.hapmap.org/index.html.
