# Supplementary material for: A genome-wide association study identifies common variants influencing serum uric acid concentrations in a Chinese population
Source: BMC Med Genomics. 2014 Feb 11;7:10. doi: 10.1186/1755-8794-7-10 (PMC3923000; doi:10.1186/1755-8794-7-10)
Supplement: Additional file 1: Table S1 — Characteristics of the subjects participated in this study. [file 1755-8794-7-10-S1.doc]

**Supplementary Table 1. Characteristics of the subjects participated in this study**

| **Variables** | **Discovery stage** | |  | **Validation stage** |
| --- | --- | --- | --- | --- |
|  | **DFTJ cohort (n=1,452)** | **FAMHES**  **(n =1,999)** |  | **(n=8,830)** |
| Age (years) | 63.0 (8.1) | 37.5 (11.1) |  | 61.9 (7.8) |
| Gender, n (%) |  |  |  |  |
| Male | 1,136 (78.2) | 1,999 (100) |  | 3,689 (41.8) |
| Female | 316 (21.8) | 0 (0) |  | 5,141 (58.2) |
| Body-mass index (kg/m²) | 24.7 (3.3) | 23.3 (3.4) |  | 24.3 (3.3) |
| Cigarette smoking, n (%) |  |  |  |  |
| Nonsmoker | 708 (48.8) | 984 (49.2) |  | 6,423 (73.3) |
| Smoker | 732 (50.2) | 1,015 (50.8) |  | 2,345 (26.7) |
| Alcohol drinking, n (%) |  |  |  |  |
| Nondrinker | 817 (56.3) | 348 (17.4) |  | 6,664 (75.5) |
| Drinker | 634 (43.7) | 1,651 (82.6) |  | 2,159 (24.5) |
| Uric acid (μmol/L) | 313.2 (86.1) | 377.5 (79.7) |  | 289.2 (80.4) |
| Data are shown as mean (SD), unless otherwise indicated. DFTJ = Dongfeng-Tongji cohort. FAMHES = Guangxi Fangchenggang Area Male Health and Examination Survey. Smokers were defined as former or current smokers; otherwise, they were viewed as nonsmokers. Drinkers were defined as former or current drinkers; otherwise, they were viewed as nondrinkers. | | | | |
|  |  |  |  |  |
|  |  |  |  |  |
|  |  |  |  |  |
