# Supplementary material for: A genome-wide association study identifies common variants influencing serum uric acid concentrations in a Chinese population
Source: BMC Med Genomics. 2014 Feb 11;7:10. doi: 10.1186/1755-8794-7-10 (PMC3923000; doi:10.1186/1755-8794-7-10)
Supplement: Additional file 2: Figure S1 — Q-Q plots for QTL analyses. [file 1755-8794-7-10-S2.doc]

**Supplementary Figure 1. Q-Q plots for QTL analyses.** The horizontal axis shows -log10 transformed expected *P* values, while the vertical axis indicates -log10 transformed observed *P* values. The genomic inflation factor λ for analysis is shown below.


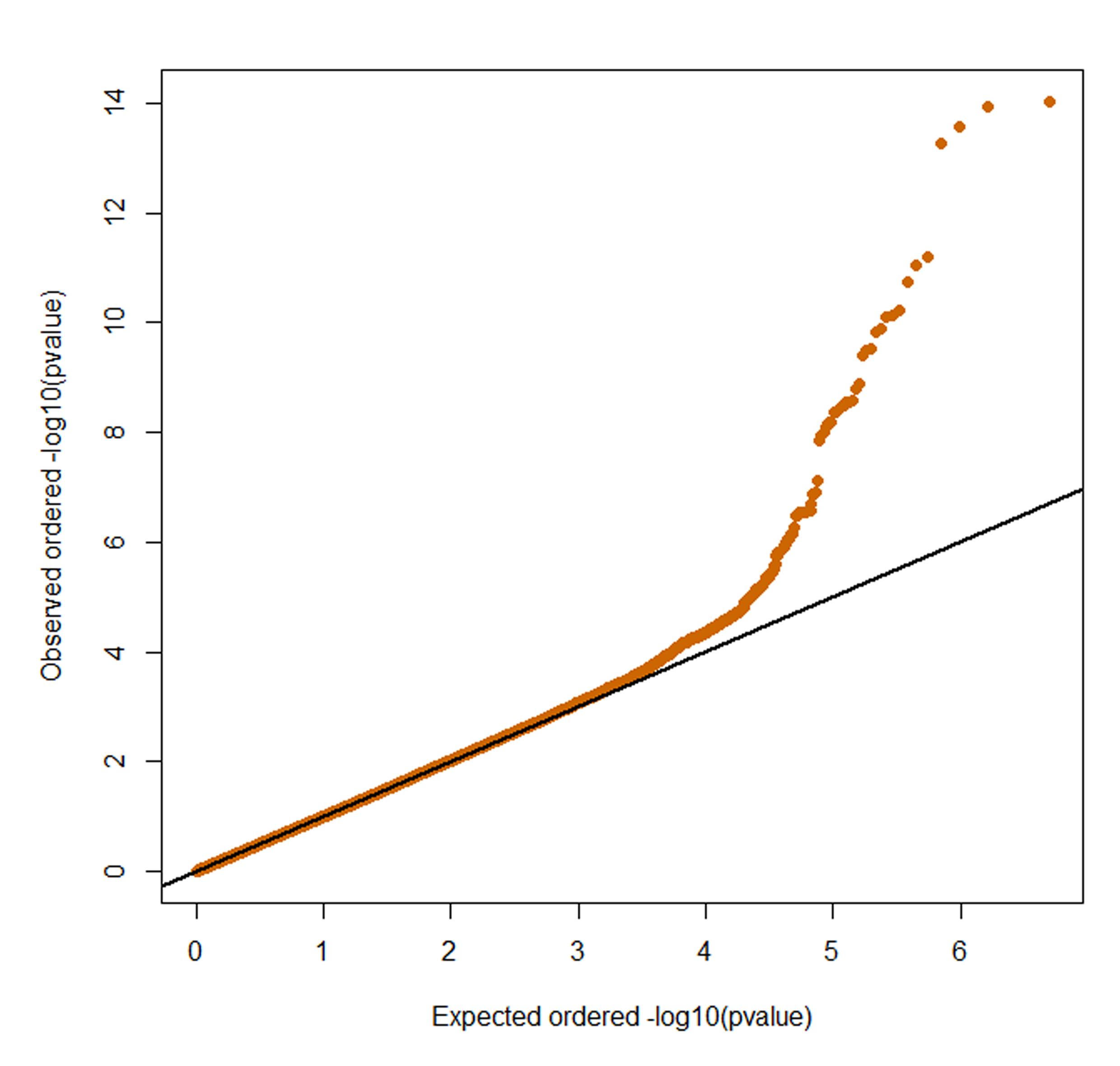


Lambda=1.007
