# Supplementary material for: A genome-wide association study identifies common variants influencing serum uric acid concentrations in a Chinese population
Source: BMC Med Genomics. 2014 Feb 11;7:10. doi: 10.1186/1755-8794-7-10 (PMC3923000; doi:10.1186/1755-8794-7-10)
Supplement: Additional file 3: Figure S2 — Associations of SNPs in Table 2 with serum uric acid concentrations. [file 1755-8794-7-10-S3.doc]

**Supplementary Figure 2. Regional plots of loci associated with serum uric acid concentrations.** The horizontal axis shows the chromosomal positions in the NCBI build 36 genome sequence. The lower part of each figure shows a LD map of D’ values drawn by the Haploview software (http://www.broadinstitute.org/haploview/haploview) using release 24 Hap Map genotype data of Han Chinese in Beijing (CHB). Black arrows indicate the direction of transcription. The lavender-filled diamonds represent LD with the SNPs in Table 2, (A) rs12129861 (B) rs11264341 (C) rs17050272 (D) rs780094 (E) rs2544390 (F) rs6770152 (G) rs11722228, rs16890979 and rs734553 (H) rs2231142 ,rs3114018,rs4148152 and rs4148155 (I) rs17632159 (J) rs729761 (K) rs742132, rs1183201 and rs1165205 (L) rs1178977 (M) rs17786744 (N) rs2941484 (O) rs10821905 (P) rs17300741 , rs505802 and rs506338 (Q) rs1394125 (R) rs6598541 (S) rs7193778 (T) rs7188445 and (U) rs7224610.

**
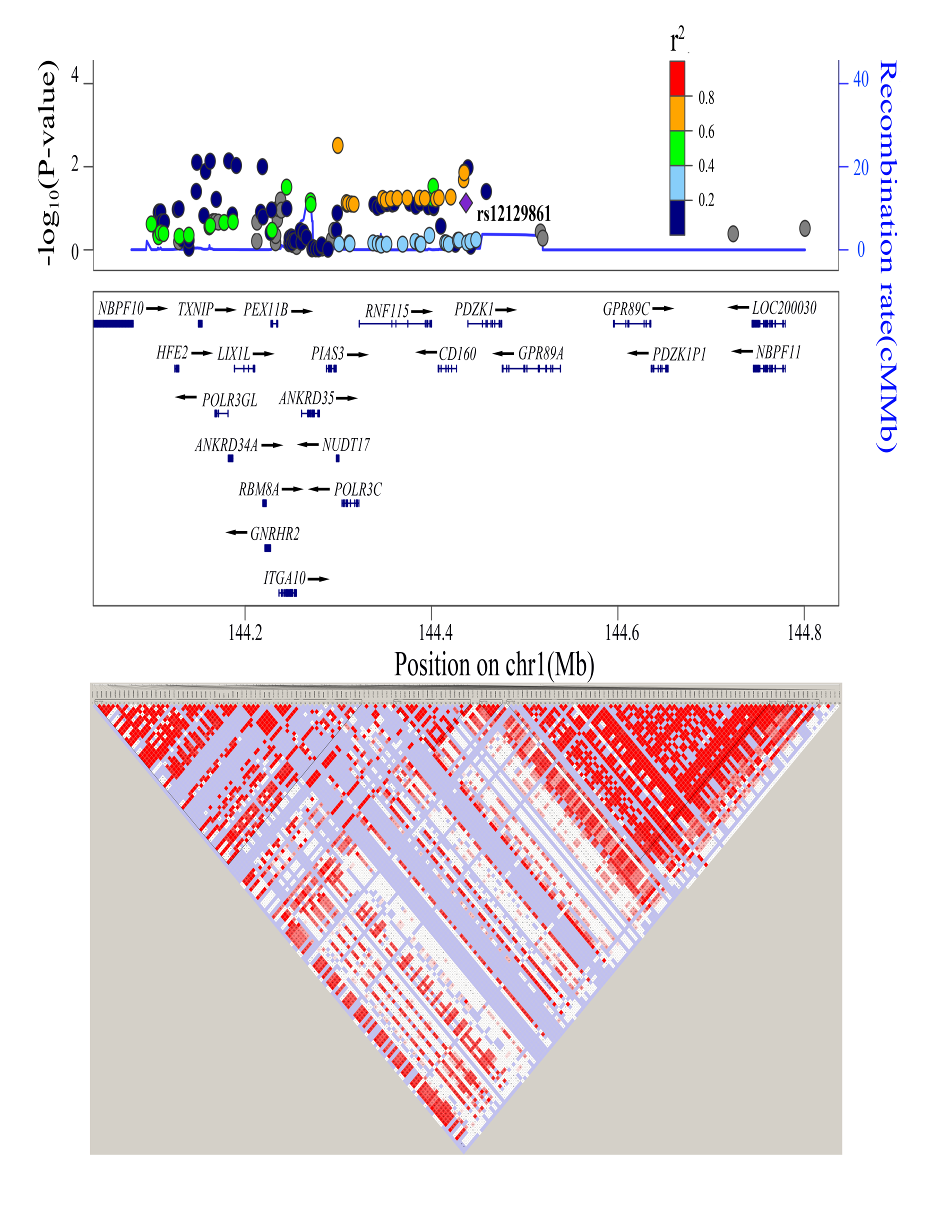

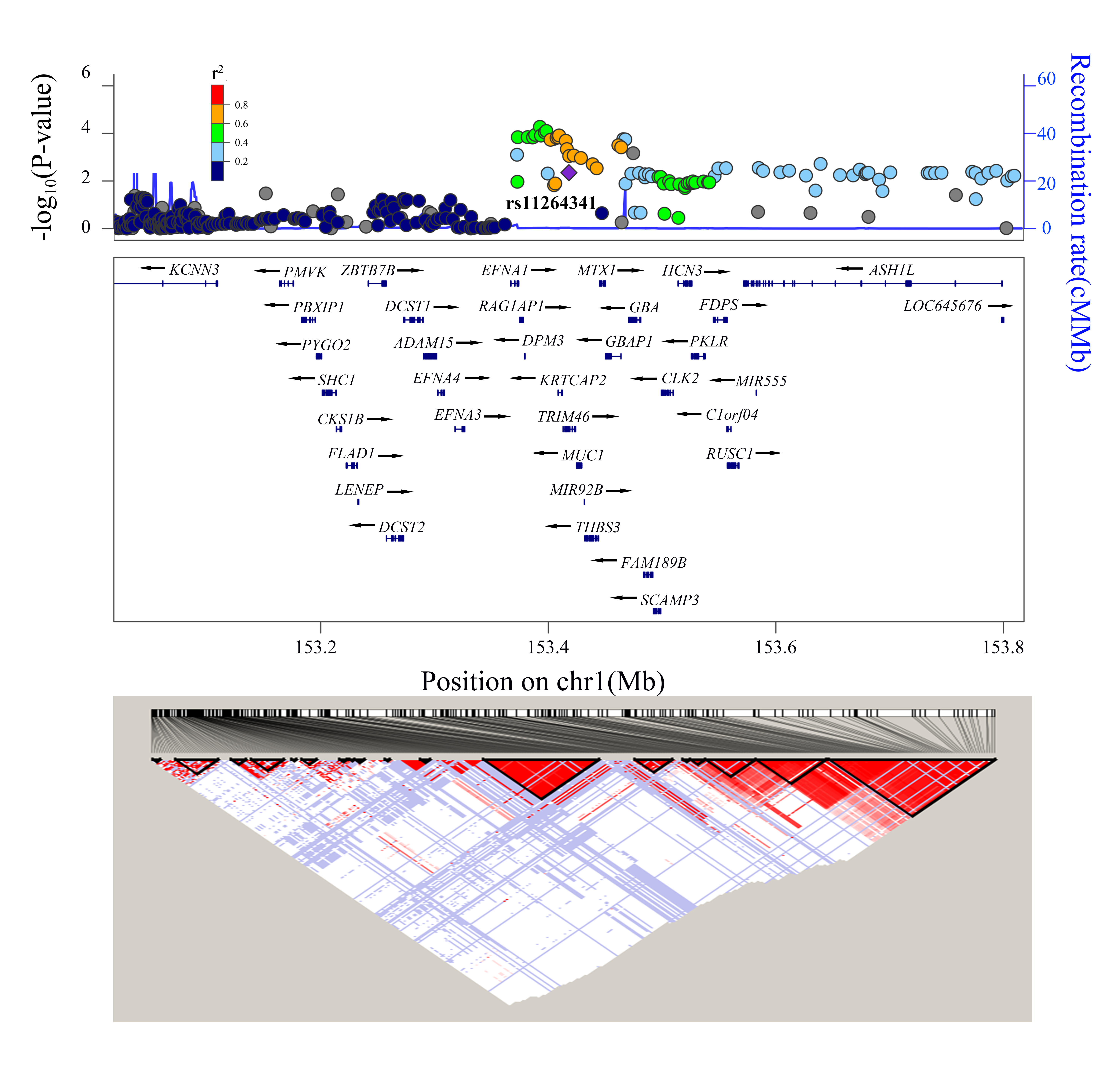
**

A

B

**
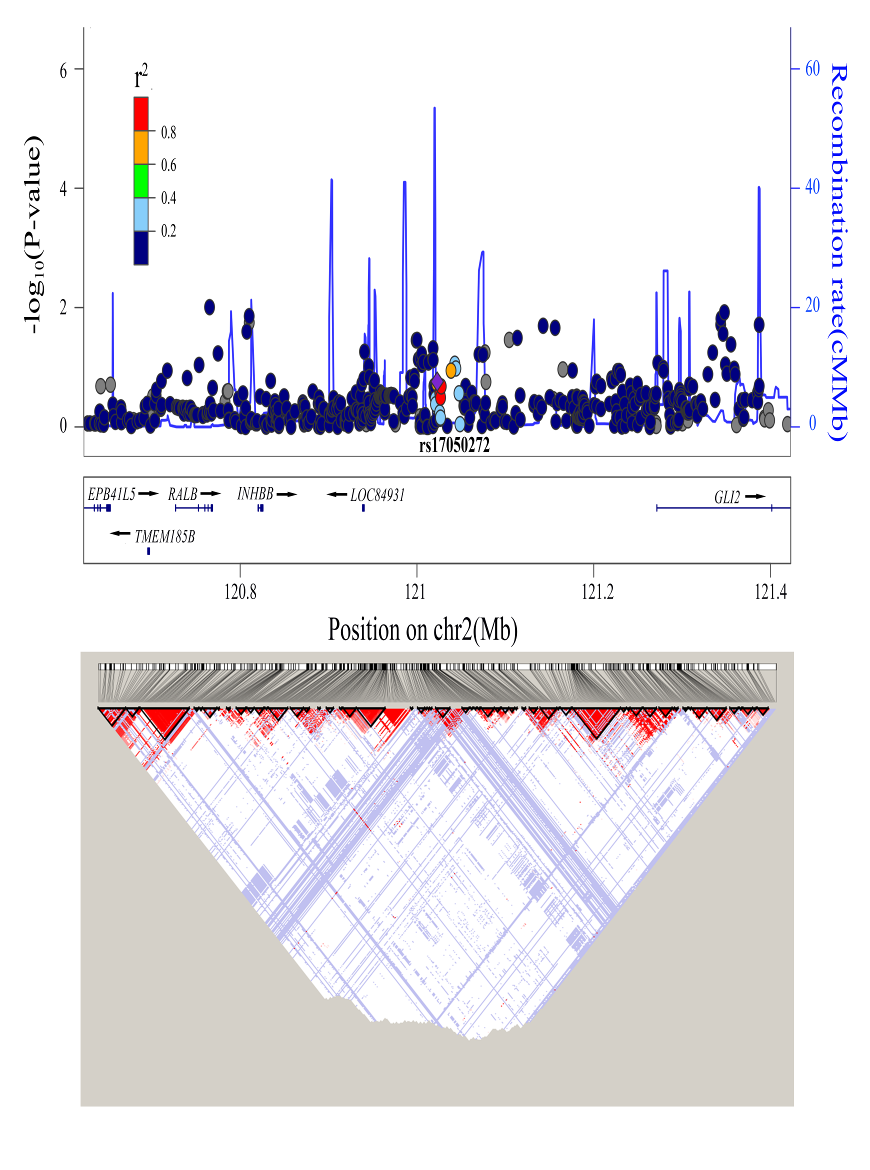

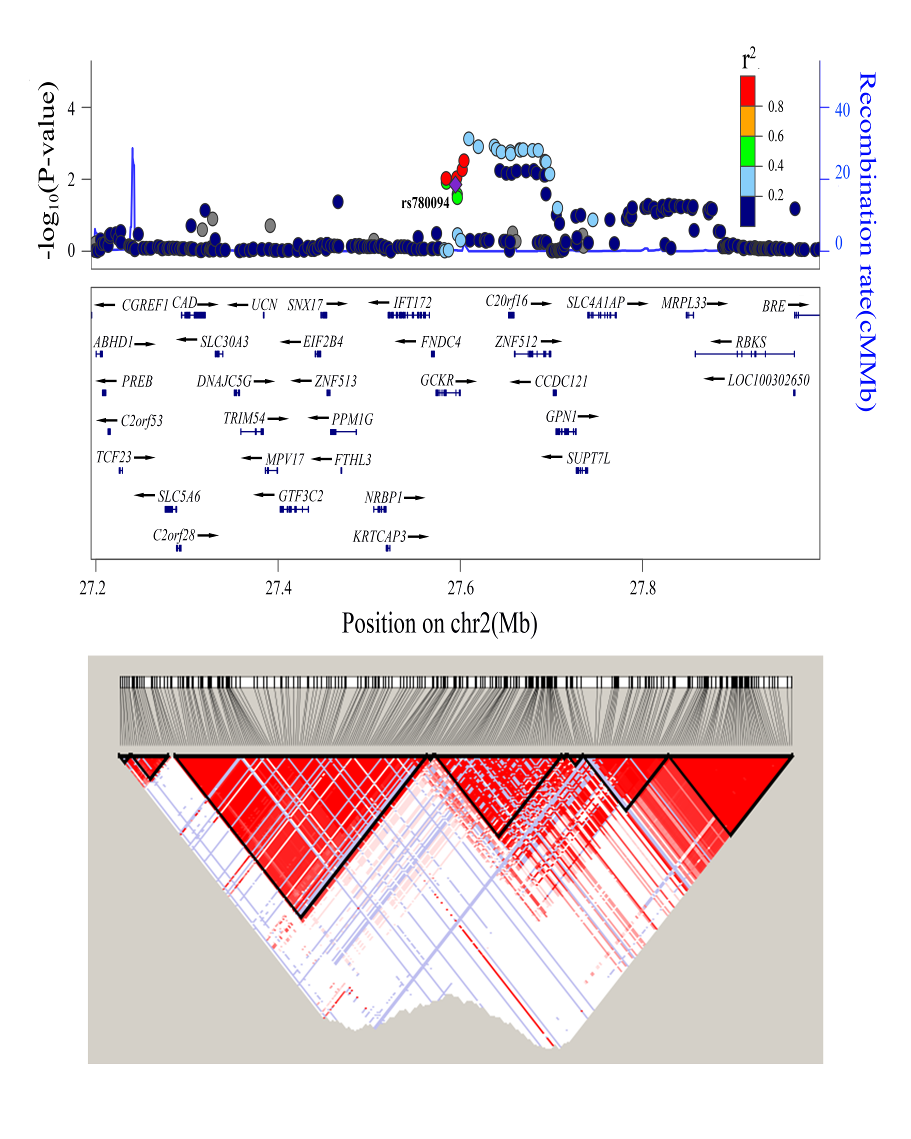
**

D

C

**
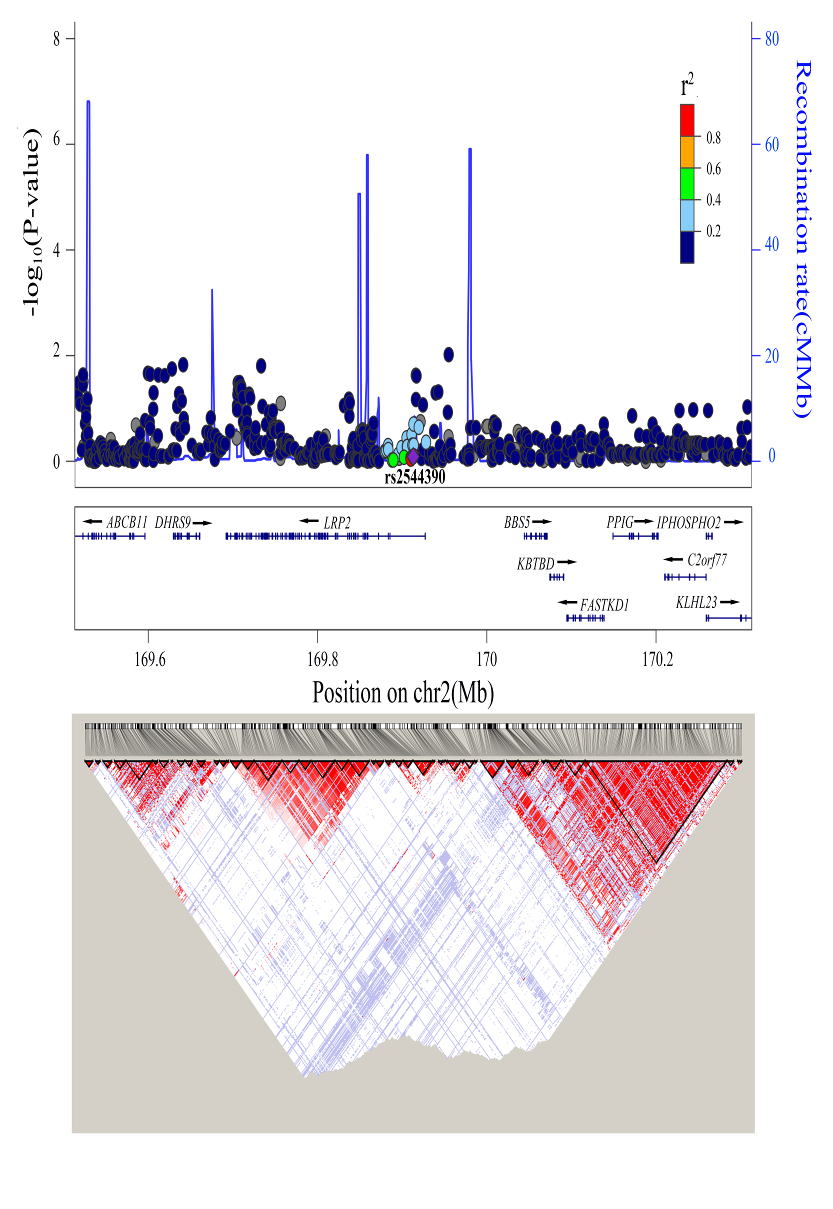
**
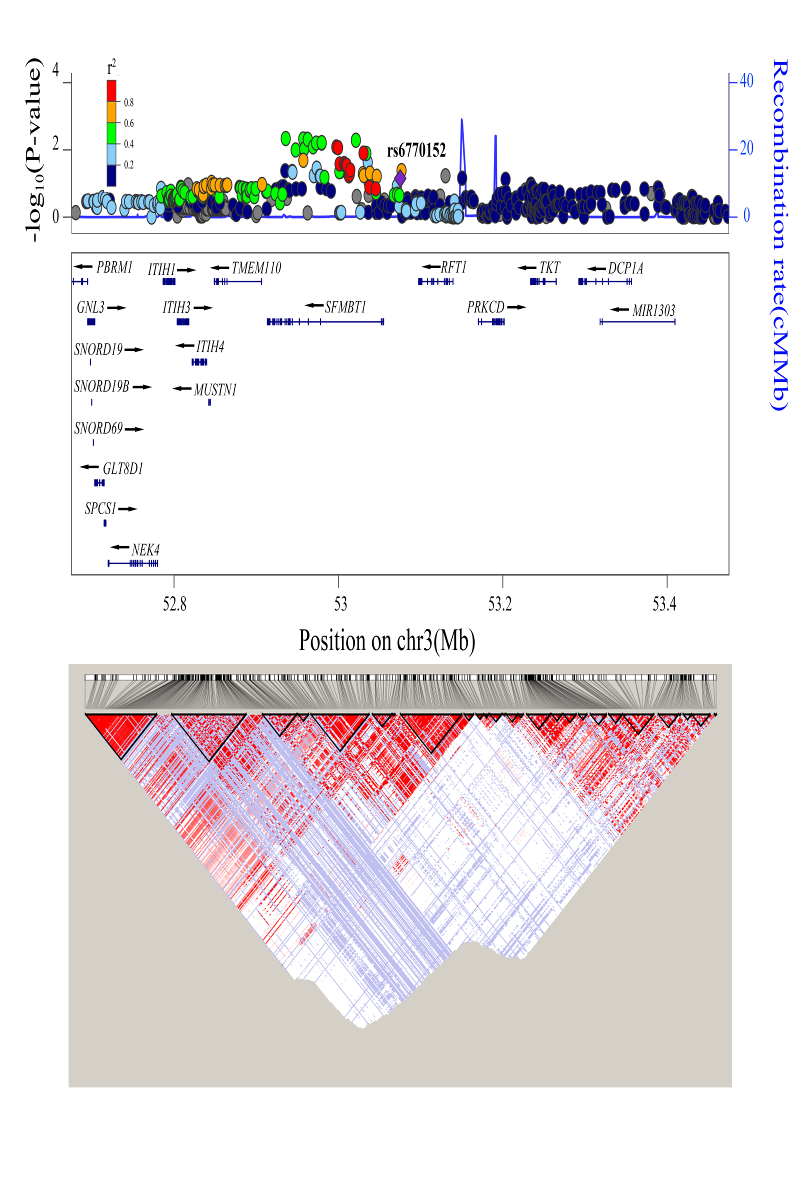


F

E


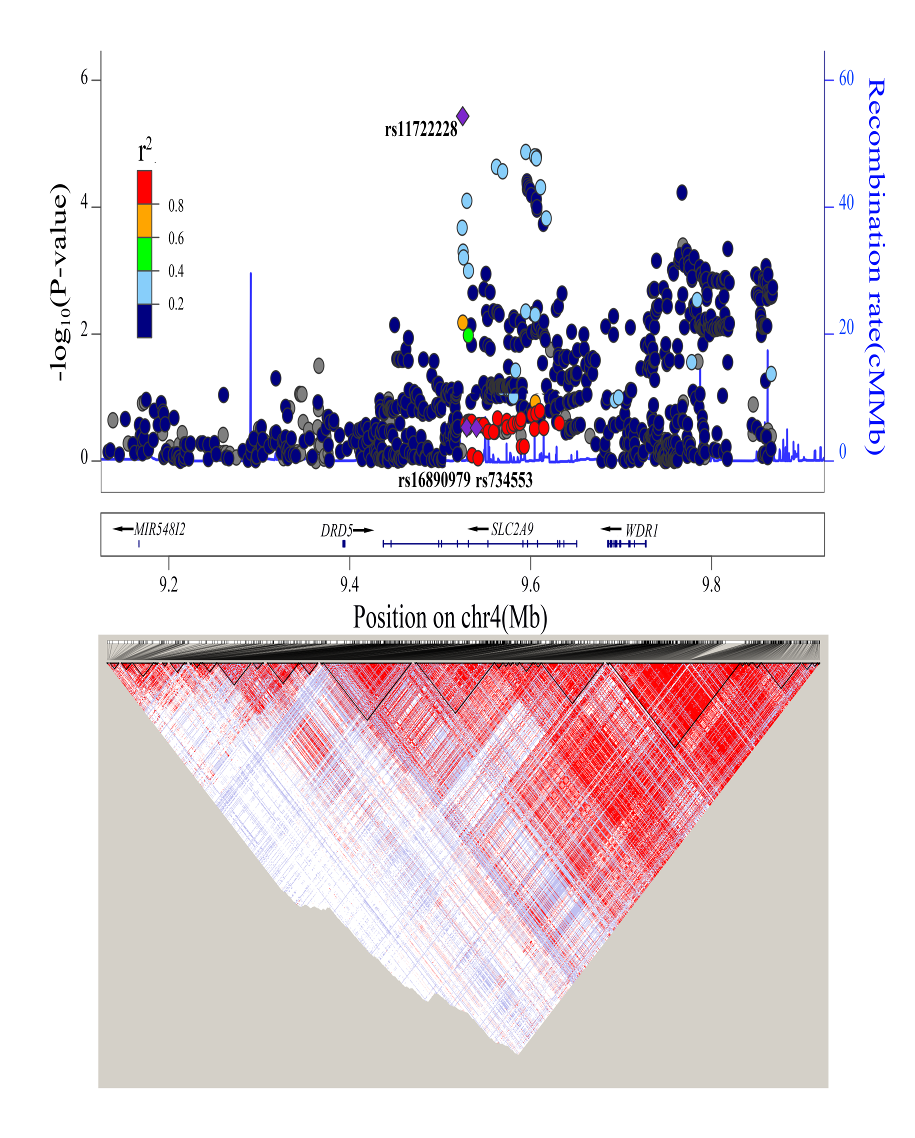

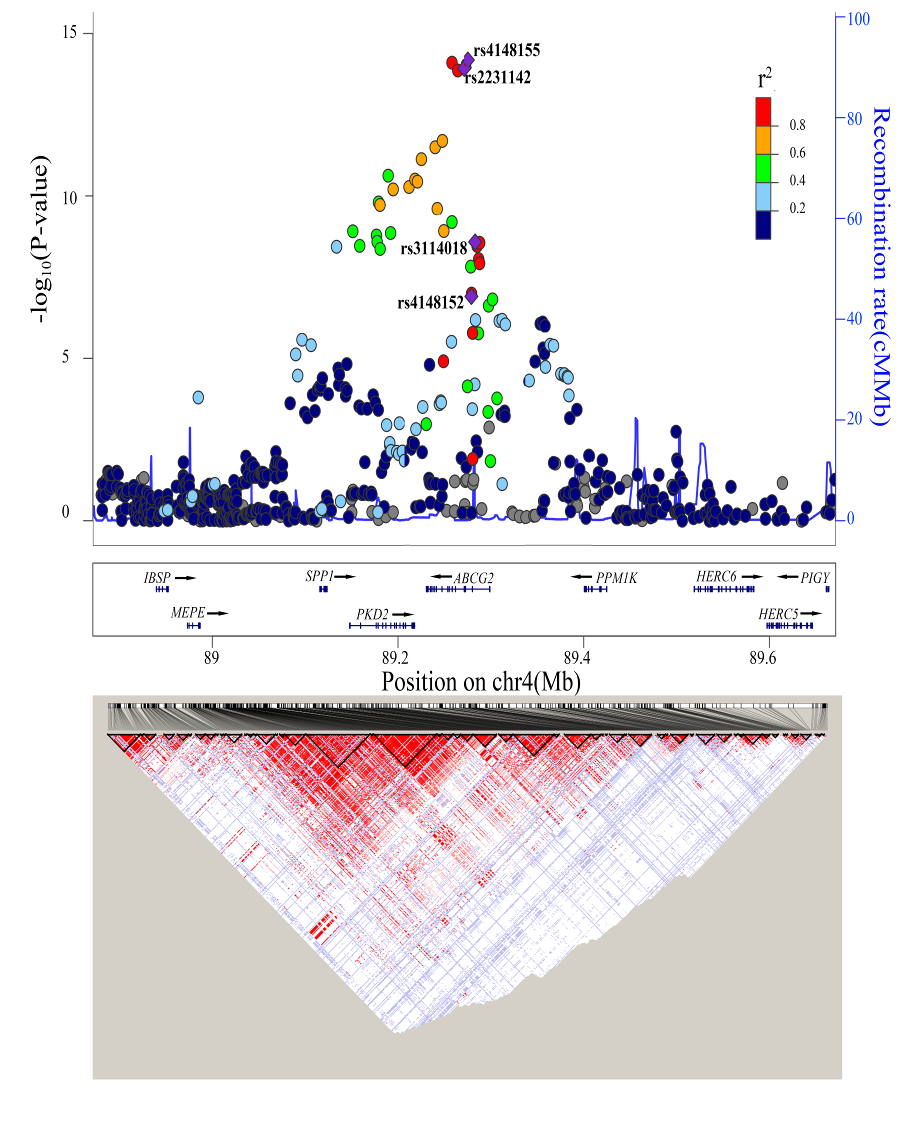


H

G


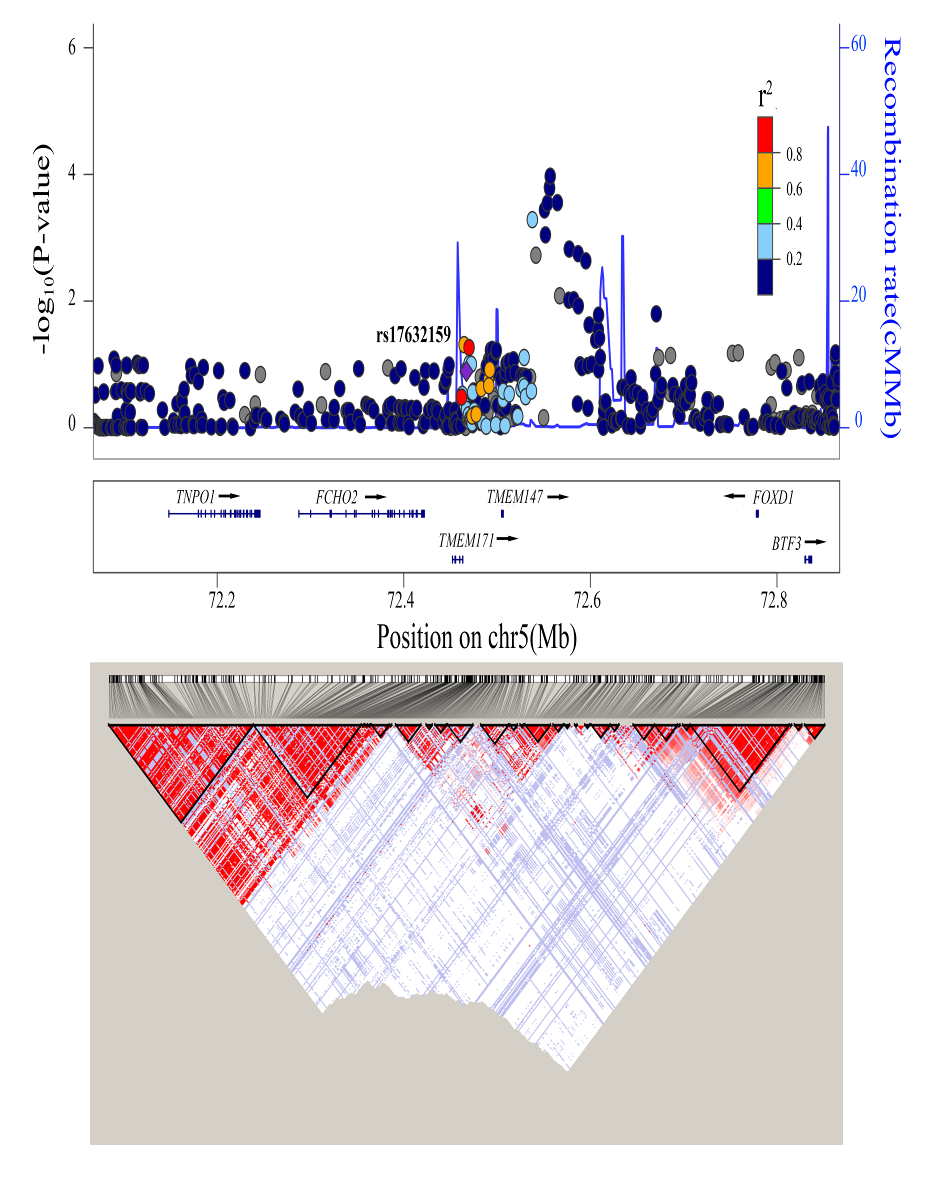

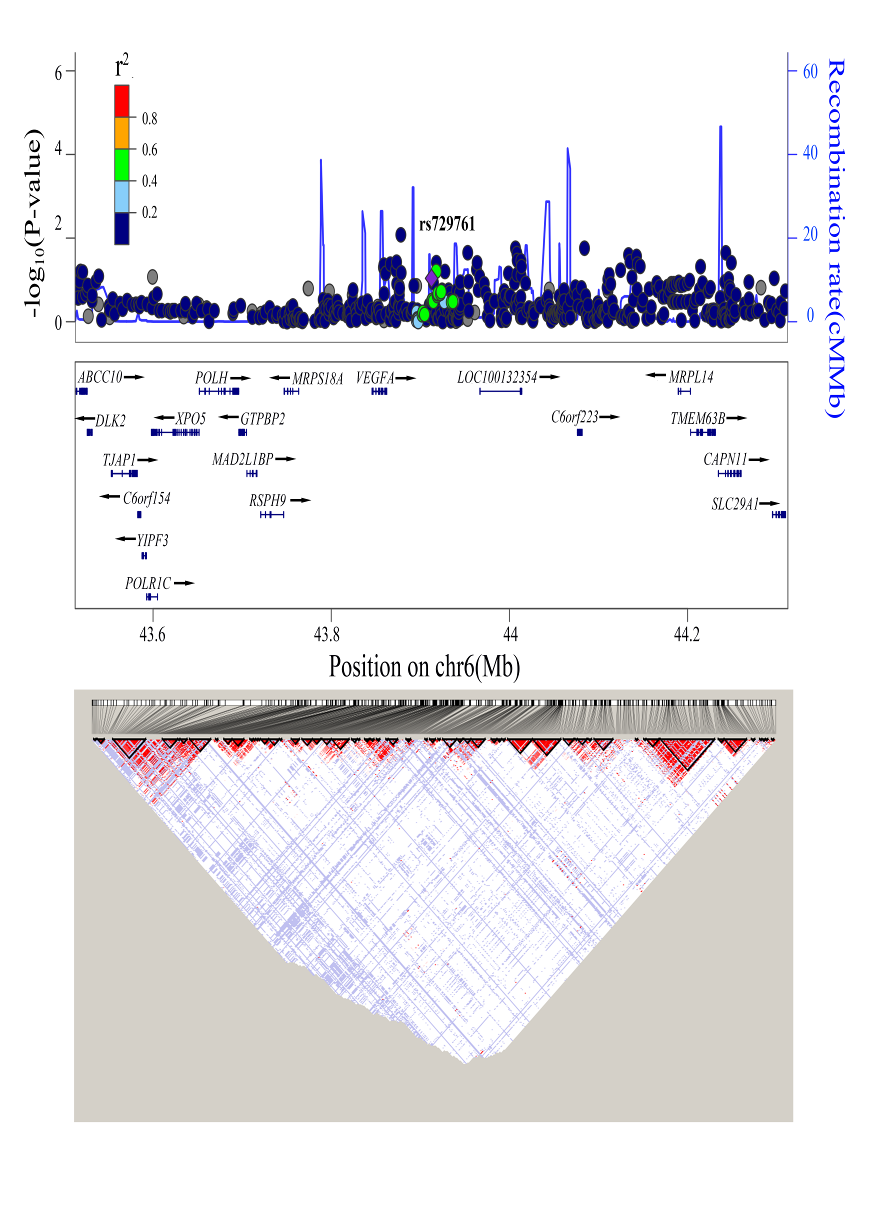


J

I


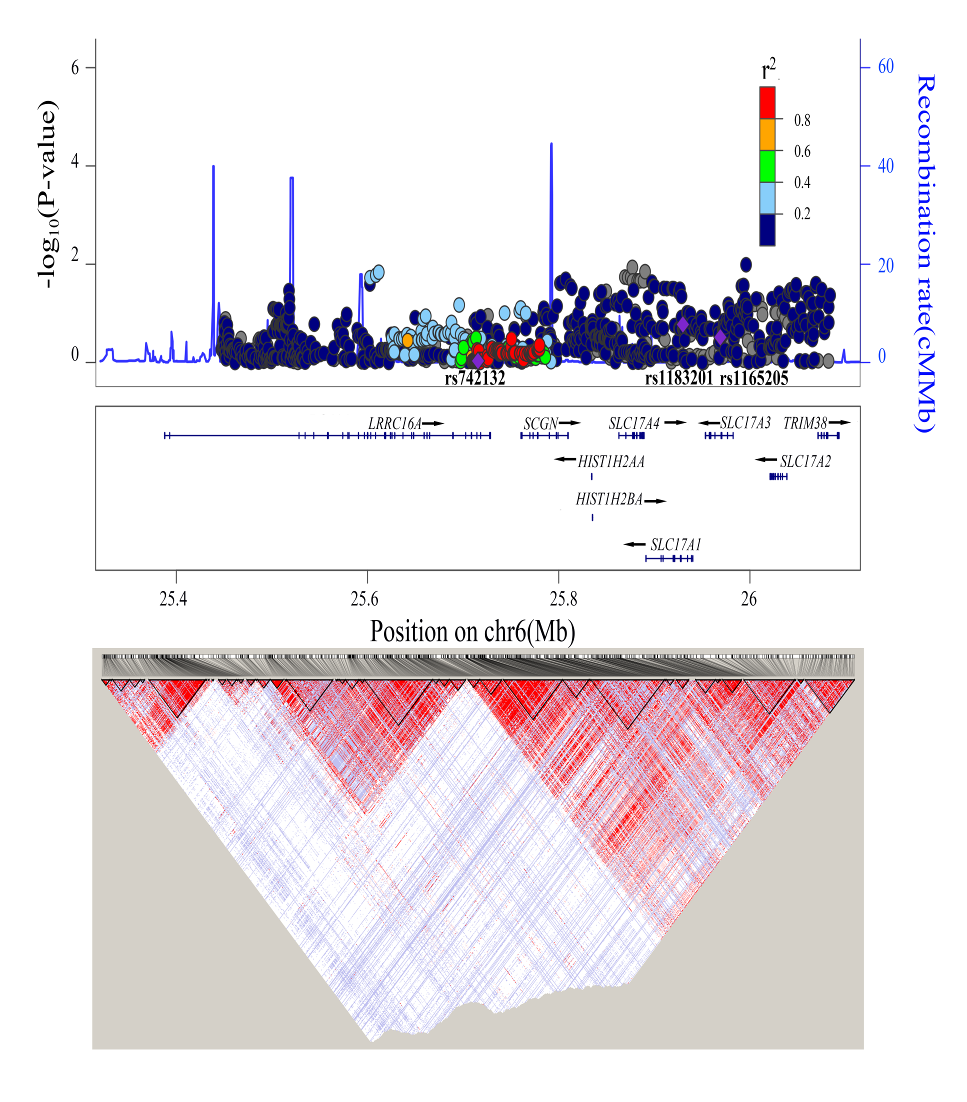

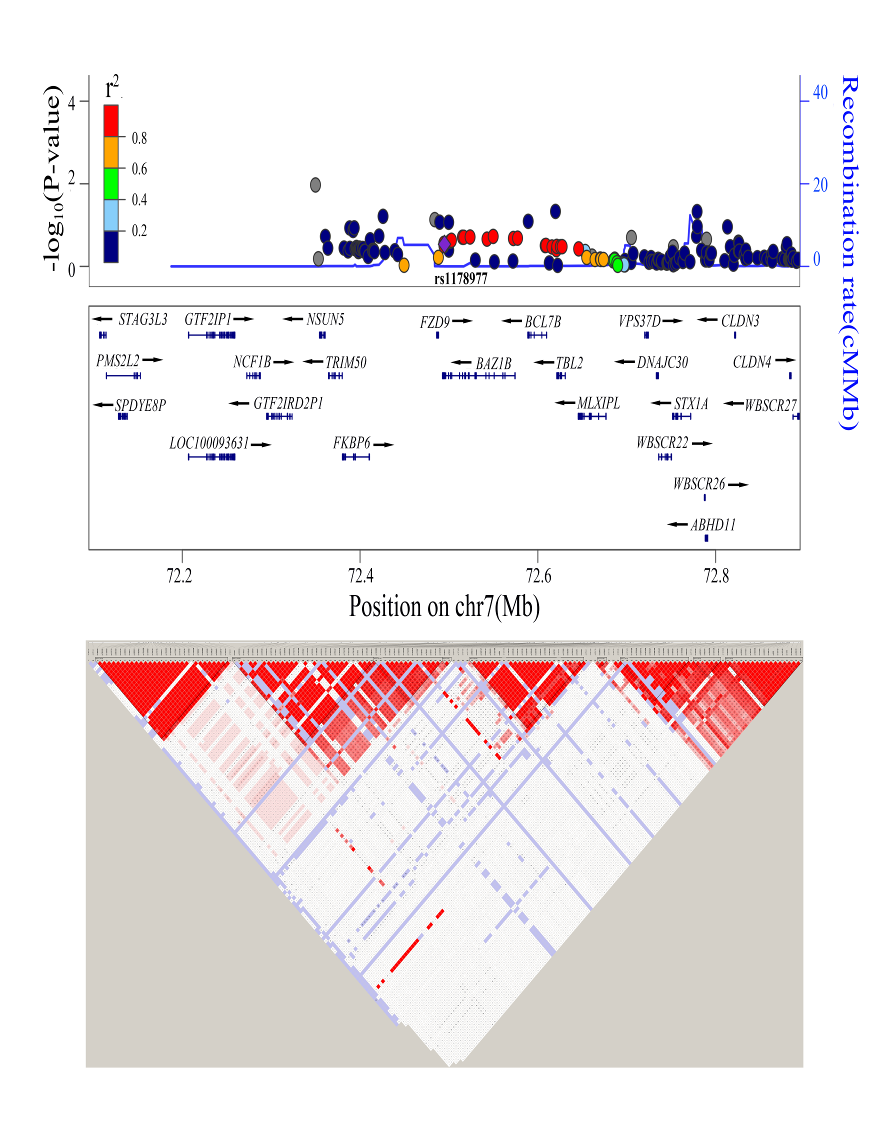


L

K


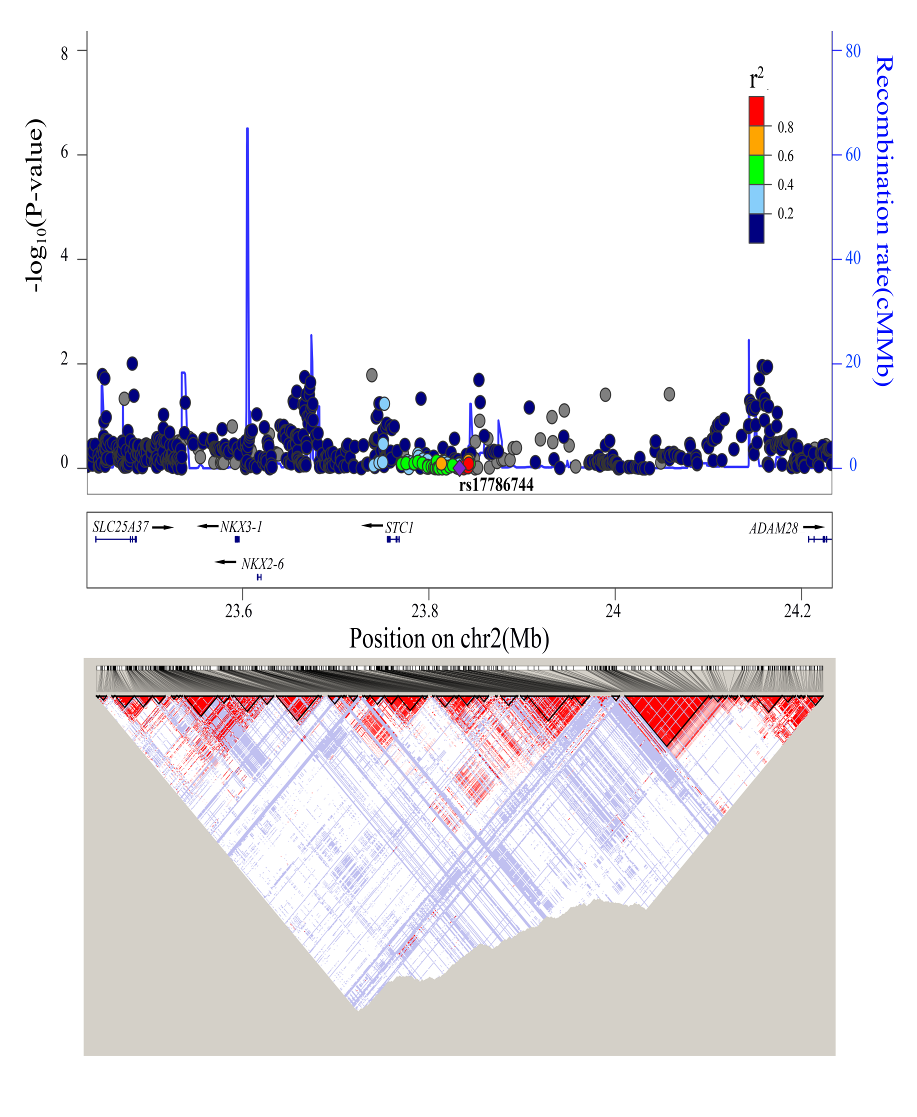

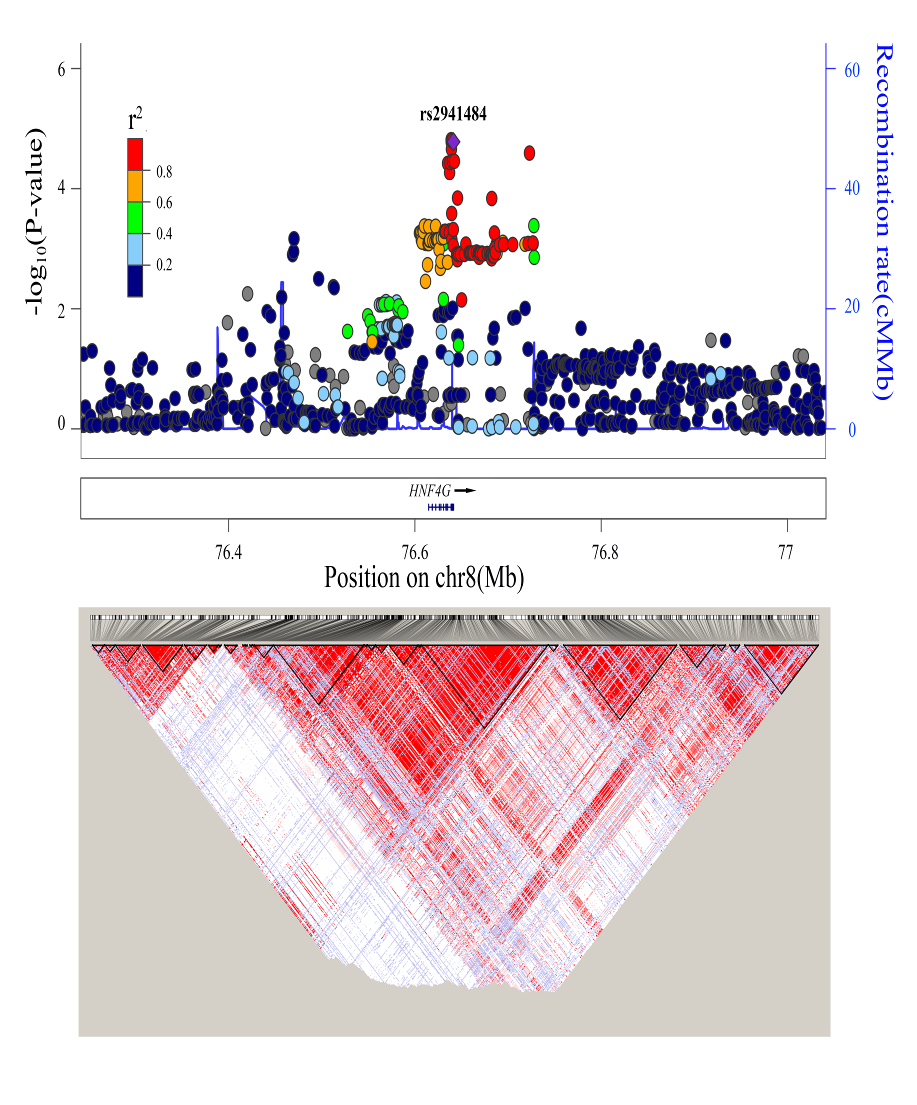


M

N


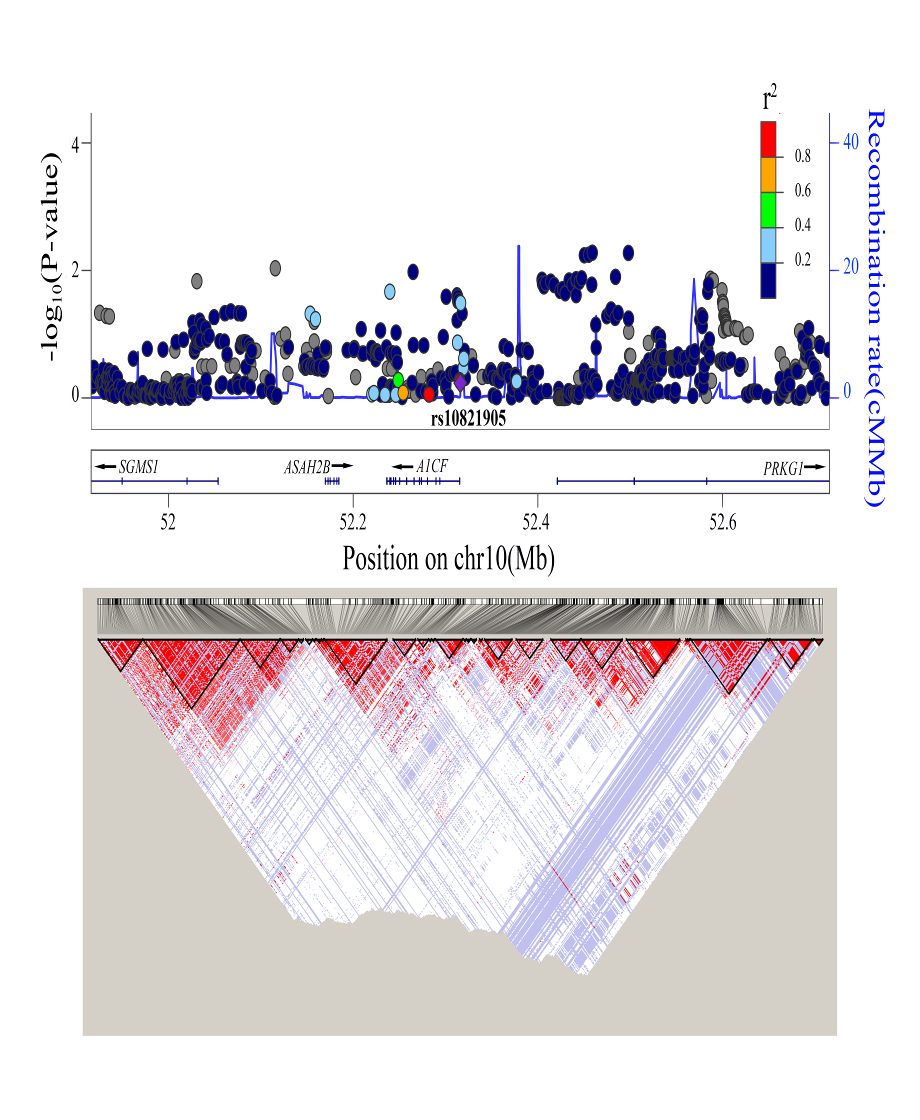

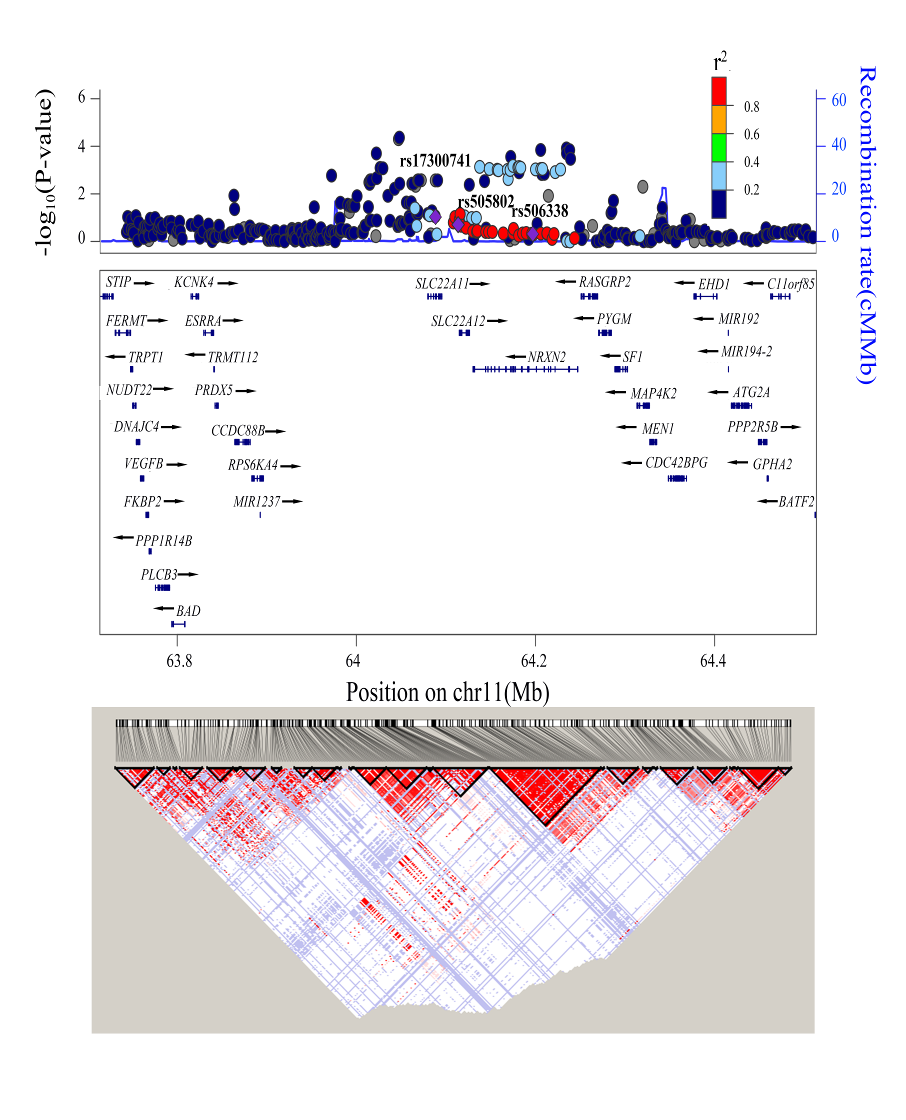


P

O


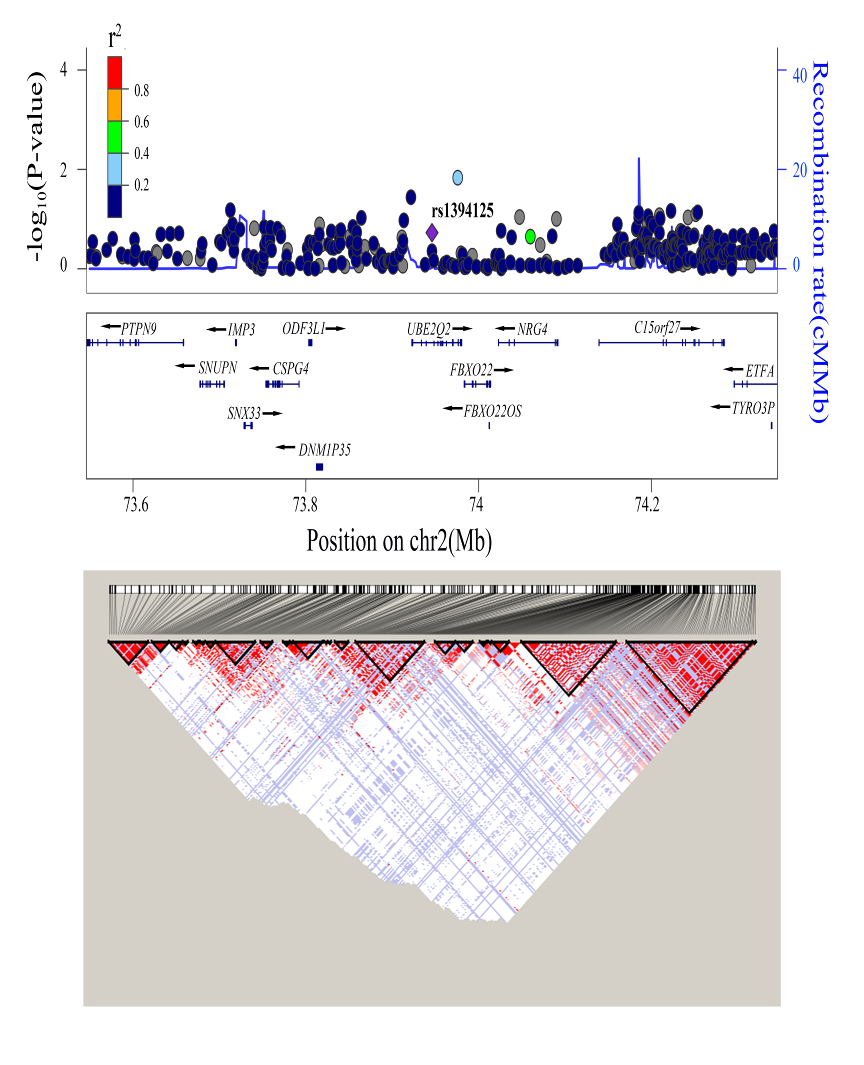

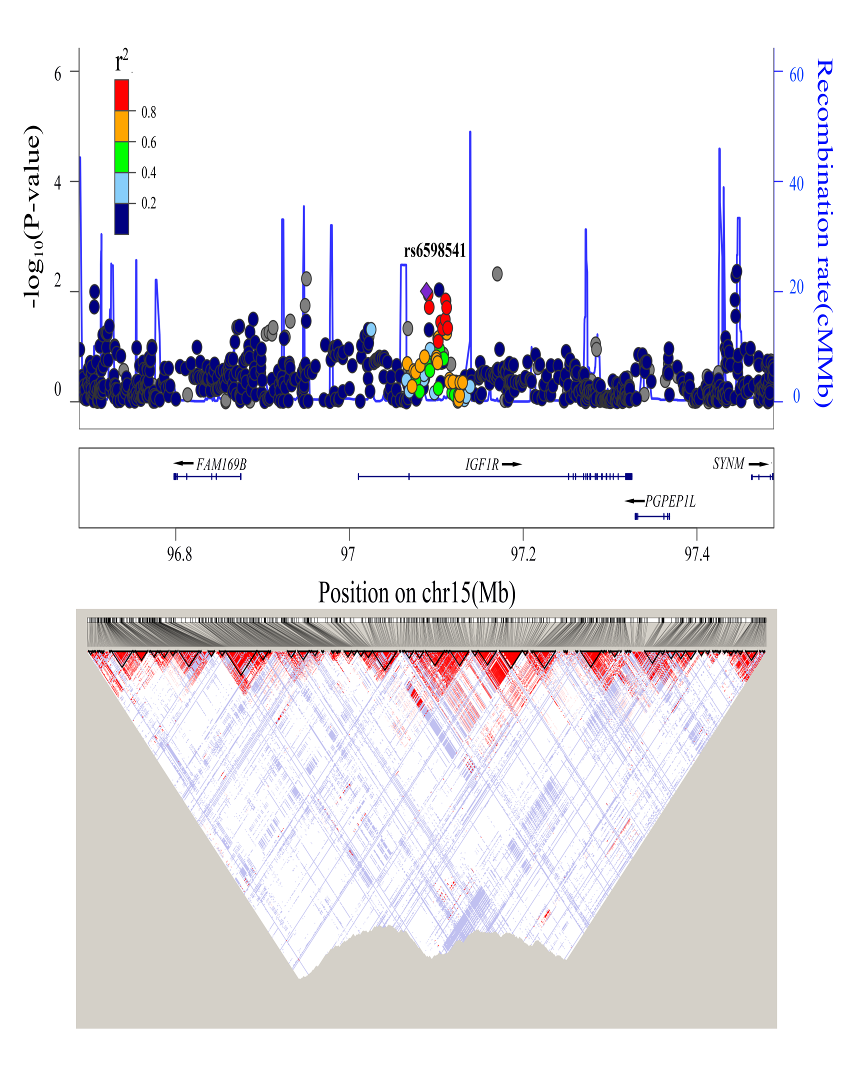


R

Q


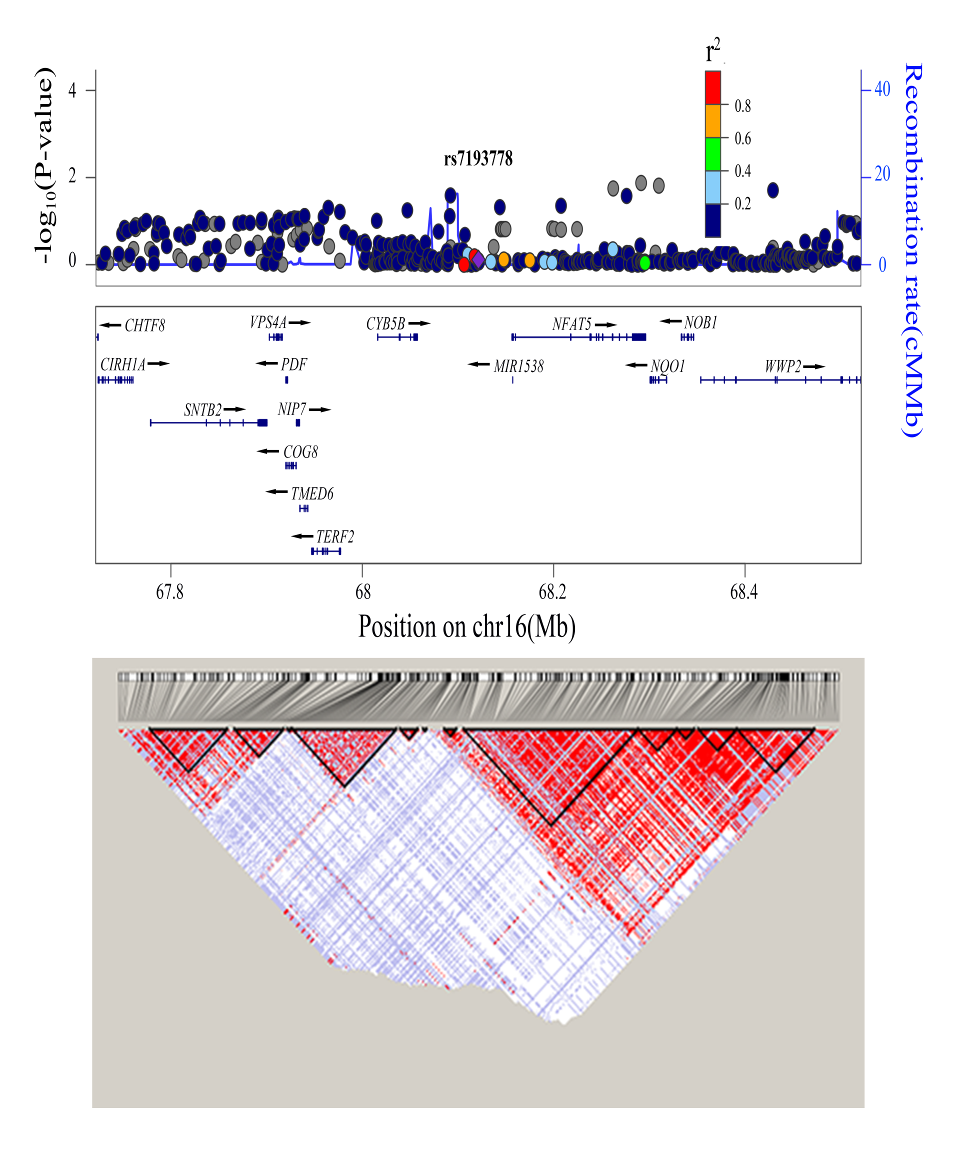

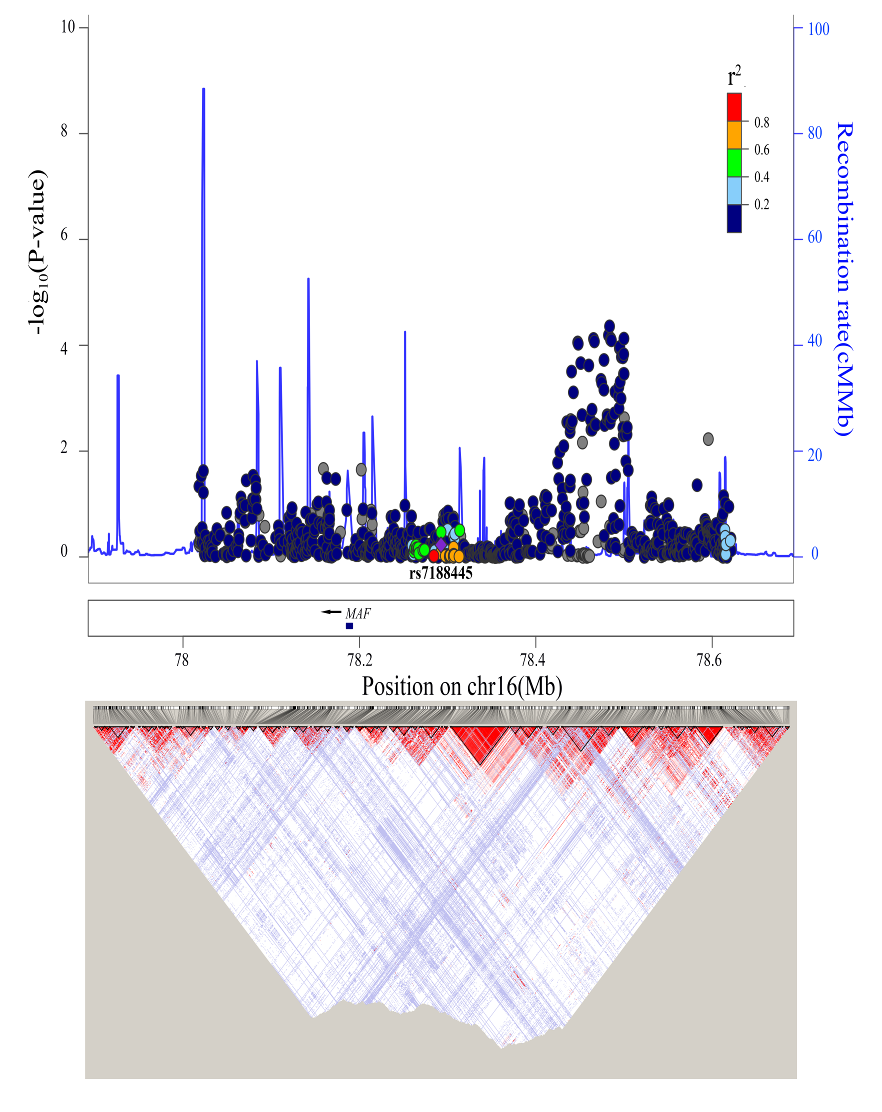


T

S


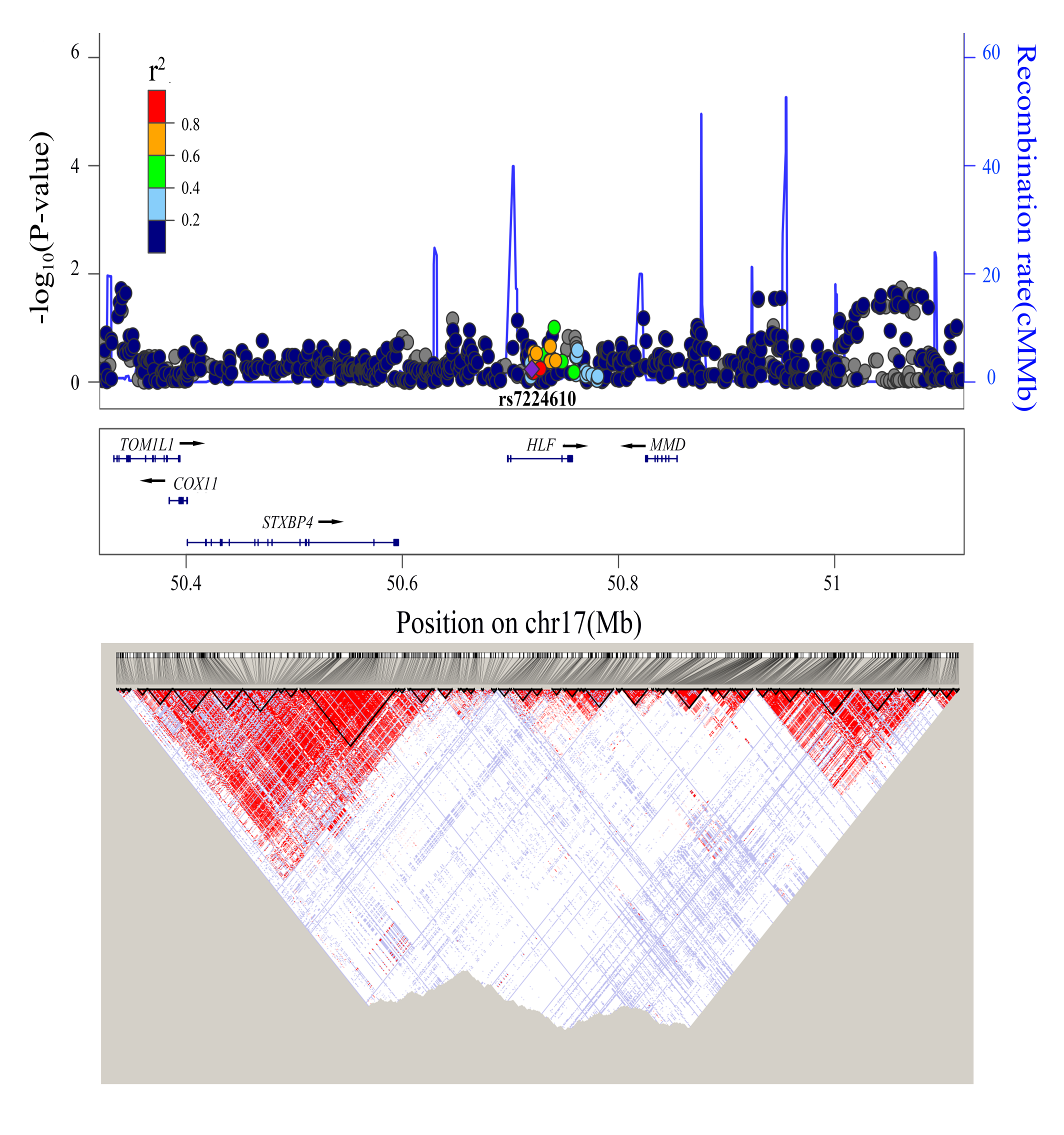


U
