# Supplementary material for: A genome-wide association study identifies common variants influencing serum uric acid concentrations in a Chinese population
Source: BMC Med Genomics. 2014 Feb 11;7:10. doi: 10.1186/1755-8794-7-10 (PMC3923000; doi:10.1186/1755-8794-7-10)
Supplement: Additional file 4: Table S2 — SNPs failed to be validated in the validation stage in the present study. [file 1755-8794-7-10-S4.doc]

**Supplementary Table 2. SNPs failed to be validated in the validation stage in the present study**

| **Gene** | **SNP** | **Chr** | **Position (bp)** | **Location** | **GWAS** | | |  | **Validation (n=3,456)** |
| --- | --- | --- | --- | --- | --- | --- | --- | --- | --- |
|  |  |  |  |  | **MAF** | **Effect size** | ***P^a^* value** |  | ***P^a^* value** |
| *SEC22B* | rs12406945 | 1 | 143814366 | intron | 0.19 | -0.019 | 9.31×10^-6^ |  | 0.917 |
| *CENTG2* | rs11899677 | 2 | 236697575 | 3utr | 0.18 | 0.013 | 9.40×10^-6^ |  | 0.809 |
| *TET2* | rs10008015 | 4 | 106224695 | intergene | 0.21 | 0.012 | 9.98×10^-6^ |  | 0.577 |
| *VEGFC* | rs437056 | 4 | 177672950 | intergene | 0.01 | 0.014 | 9.77×10^-6^ |  | 0.548 |
| *TNFRSF11B* | rs3134053 | 8 | 120015321 | intron | 0.12 | 0.021 | 3.12×10^-6^ |  | 0.691 |
| LOC100286746 | rs12056908 | 8 | 120074413 | intergene | 0.07 | 0.018 | 3.52×10^-6^ |  | 0.273 |
| Chr, chromosome. MAF, minor allele frequency calculated using the data from all the subjects in the analysis. Effect size, represents the effect of a minor allele on the standardized trait (estimated coefficient of the term for the number of the minor alleles). The NCBI build 36 was used as the reference genome.  ^a^ Linear regression analysis adjusted for age, sex, body mass index (BMI), cigarette smoking and alcohol drinking | | | | | | | | | |
