# Supplementary material for: A genome-wide association study identifies common variants influencing serum uric acid concentrations in a Chinese population
Source: BMC Med Genomics. 2014 Feb 11;7:10. doi: 10.1186/1755-8794-7-10 (PMC3923000; doi:10.1186/1755-8794-7-10)
Supplement: Additional file 5: Table S3 — Covariates and SNPs in relation to serum uric acid levels in 10,282 individuals. [file 1755-8794-7-10-S5.doc]

**Supplementary Table 3. Covariates and SNPs in relation to serum uric acid levels in 10,282 individuals**

|  | **Model 1** | | |  | **Model 2** | | |
| --- | --- | --- | --- | --- | --- | --- | --- |
| **Variables** | **Beta (s.e.)** | ***P* value** | **r^2^ (%)** |  | **Beta (s.e.)** | ***P* value** | **r^2^ (%)** |
| Gender | -0.184(0.0071) | 2.88×10^-145^ | 16.86 |  | -0.171(0.0008) | 6.71×10^-95^ | 16.21 |
| Age | 0.005(0.0003) | 2.51×10^-50^ | 1.71 |  | 0.006(0.0004) | 1.01×10^-50^ | 2.14 |
| BMI | 0.018(0.0007) | 7.15×10^-125^ | 4.69 |  | 0.018(0.0008) | 3.50×10^-96^ | 4.45 |
| Smoking | 0.015(0.0074) | 0.04 | 0.05 |  | 0.020(0.0086) | 0.02 | 0.07 |
| Drinking | 0.007(0.0068) | 0.34 | 0.01 |  | 0.004(0.0078) | 0.58 | 0.01 |
| rs11722228 | -- | -- | -- |  | 0.043(0.0042) | 1.19×10^-24^ | 1.03 |
| rs2231142 | -- | -- | -- |  | 0.040(0.0048) | 1.91×10^-16^ | 1.09 |
| rs3114018 | -- | -- | -- |  | -0.006(0.0059) | 0.34 | 0.01 |
| rs4148152 | -- | -- | -- |  | -0.009(0.0061) | 0.15 | 0.07 |

The NCBI build 36 was used as the reference genome.
