# Supplementary material for: A genome-wide association study identifies common variants influencing serum uric acid concentrations in a Chinese population
Source: BMC Med Genomics. 2014 Feb 11;7:10. doi: 10.1186/1755-8794-7-10 (PMC3923000; doi:10.1186/1755-8794-7-10)
Supplement: Additional file 6: Table S4 — Interaction between SNPs and gender, BMI, alcohol drinking and cigarette smoking. [file 1755-8794-7-10-S6.doc]

**Supplementary Table 4. Interaction between SNPs and gender, BMI, alcohol drinking and cigarette smoking**

|  |  | **Serum uric acid(μmol/L)** | | |  | **Log (SUA)** | |  | **Log(SUA)** |
| --- | --- | --- | --- | --- | --- | --- | --- | --- | --- |
| **SNP** | **Variation** | **AA** | **Aa** | **aa** |  | **β(SE)** | ***P*-value** **^a^** |  | ***P*-for interaction** **^b^** |
| rs11722228 (CC/CT/TT) |  |  |  |  |  |  |  |  |  |
|  | Gender |  |  |  |  |  |  |  |  |
|  | Male | 321.24(78.94),n=2277 | 331.65(79.06),n=2061 | 344.97(84.03),n=454 |  | 0.035(0.005) | 3.09×10^-11^ |  | **0.040** |
|  | Female | 252.06(67.00),n=2554 | 267.90(69.92),n=2316 | 275.60(74.57),n=554 |  | 0.051(0.005) | 3.74×10^-21^ |  |
|  | BMI |  |  |  |  |  |  |  |  |
|  | BMI<24 | 267.35(76.78),n=2321 | 279.80(76.31),n=1972 | 286.53(74.51),n=494 |  | 0.046(0.006) | 3.34×10^-16^ |  | 0.606 |
|  | BMI>=24 | 301.52(81.08),n=2457 | 313.16(81.49),n=2353 | 326.92(92.15),n=502 |  | 0.041(0.005) | 5.17×10^-15^ |  |
|  | Alcohol drinking |  |  |  |  |  |  |  |  |
|  | Nondrinker | 273.63(77.61),n=3520 | 289.07(78.98),n=3179 | 298.38(85.13),n=744 |  | 0.049(0.004) | 8.49×10^-28^ |  | **0.016** |
|  | Drinker | 314.37(81.01),n=1314 | 321.33(81.16),n=1198 | 330.57(84.87),n=262 |  | 0.029(0.007) | 4.81×10^-5^ |  |
|  | Cigarette smoking |  |  |  |  |  |  |  |  |
|  | Nonsmoker | 270.12(76.39),n=3364 | 284.64(78.99),n=3012 | 294.83(86.51),n=720 |  | 0.049(0.005) | 8.28×10^-26^ |  | **0.035** |
|  | Smoker | 319.33(79.83),n=1440 | 328.38(76.68),n=1335 | 337.01(77.65),n=282 |  | 0.031(0.007) | 1.87×10^-6^ |  |
| rs2231142 (GG/TG/TT) |  |  |  |  |  |  |  |  |  |
|  | Gender |  |  |  |  |  |  |  |  |
|  | Male | 317.36(76.35),n=1648 | 333.85(77.43),n=1471 | 357.35(97.68),n=307 |  | 0.057(0.006) | 1.54×10^-19^ |  | **0.020** |
|  | Female | 257.63(65.57),n=2241 | 263.40(71.52),n=2075 | 278.37(74.12),n=441 |  | 0.037(0.006) | 2.04×10^-10^ |  |
|  | BMI |  |  |  |  |  |  |  |  |
|  | BMI<24 | 266.37(71.97),n=1824 | 276.89(76.99),n=1715 | 289.96(84.47),n=348 |  | 0.047(0.006) | 2.03×10^-13^ |  | 0.995 |
|  | BMI>=24 | 298.05(77.25),n=2017 | 308.10(83.31),n=1778 | 330.32(96.35),n=389 |  | 0.042(0.006) | 1.69×10^-12^ |  |
|  | Alcohol drinking |  |  |  |  |  |  |  |  |
|  | Nondrinker | 273.52(72.32),n=2913 | 284.19(80.75),n=2695 | 301.88(90.83),n=576 |  | 0.045(0.005) | 2.21×10^-19^ |  | 0.869 |
|  | Drinker | 310.92(80.67),n=978 | 319.64(79.00),n=850 | 340.26(94.62),n=172 |  | 0.019(0.006) | 7.47×10^-8^ |  |
|  | Cigarette smoking |  |  |  |  |  |  |  |  |
|  | Nonsmoker | 270.96(73.46),n=2814 | 278.41(78.84),n=2565 | 299.72(89.65),n=564 |  | 0.045(0.005) | 2.61×10^-18^ |  | 0.809 |
|  | Smoker | 315.40(74.60),n=1051 | 331.17(76.96),n=960 | 347.01(91.93),n=179 |  | 0.048(0.008) | 176×10^-9^ |  |

Note, data from combined; The NCBI build 36 was used as the reference genome. Serum UA, serum uric acid; Results with *P*-interaction <0.05 are shown in boldface. ^a^ *P*-value for the genotypes in each stratum after multivariate adjustment for covariables including age, gender, BMI, smoking, and drinking except for the stratified variable. **^b^** *P*-value for the interaction term between the genotypes and each covariable in the multivariate adjusted model
